# Effective RNA Complexation by [2]Catenanes Confers Enhanced Resistance to Enzymatic Degradation

**DOI:** 10.1002/chem.202501631

**Published:** 2025-07-24

**Authors:** Dimitri Delcourt, José García Coll, Fabien B. L. Cougnon, Sébastien Ulrich

**Affiliations:** ^1^ Department of Chemistry, Nanoscience Center University of Jyväskylä P.O. Box 35 Jyväskylä FI‐40014 Finland; ^2^ Institut des Biomolécules Max Mousseron (IBMM) Université de Montpellier, CNRS, ENSCM Montpellier France

**Keywords:** catenanes, dynamic covalent synthesis, nucleases, proteases, RNA complexation

## Abstract

Cationic [2]catenanes bearing *l*‐arginine residues were synthesized via dynamic covalent self‐assembly in water. These peptide‐based mechanically interlocked molecules (MIMs) exhibit proteolytic stability, efficiently complex small‐interfering RNA (siRNA), and protect it from nuclease degradation. Their performance in siRNA binding is attributed to multivalency and preorganization enforced by the mechanical bond.

## Graphical abstract

Peptide‐based cationic [2]catenanes are resistant to proteases and efficiently complex siRNA, providing protection from degradation by nucleases.

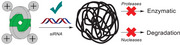

## 1 Introduction

Advances in nucleic acid‐based therapeutics for cancer and infectious diseases are strongly correlated to the development of delivery vectors capable of safely transporting active agents to their site of action. These artificial vectors must efficiently and dynamically complex nucleic acids,^[^ ^1^, ^2^, ^3^, ^4^ ^]^ while remaining stable in biological media and resisting enzymatic degradation by nucleases and proteases.^[^ ^5^ ^]^

Nature offers a compelling blueprint for the design of artificial vectors. In the nucleosome, reversible DNA compaction is achieved through the self‐assembly of histone proteins into octamers that present multiple protruding cationic residues responsible for DNA recognition.^[^ ^6^ ^]^ Multivalent cationic clusters have thus emerged for nucleic acid complexation,^[^ ^7^, ^8^ ^]^ and cationic polymers, dendrimers,^[^ ^9^, ^10^, ^11^ ^]^ and peptides^[^ ^12^ ^]^ have gained popularity due to their high efficacy and relative ease of synthesis. The three‐dimensional organization of cationic domains is expected to play a pivotal role in the complexation of nucleic acids, in the same way the spatial arrangement of cationic residues in cell‐penetrating peptides influences membrane translocation.^[^ ^13^ ^]^ The Ulrich group showed that preorganized cyclic cationic peptides complex DNA more effectively than their linear analogues.^[^ ^14^, ^15^ ^]^ This preorganization can potentially be further enhanced through the incorporation of mechanical bonds.^[^ ^16^ ^]^ Compared to their topologically trivial counterparts, mechanically interlocked molecules (MIMs)^[^ ^17^, ^18^ ^]^ possess a compact, entangled core that can improve resistance to enzymatic degradation and offer multivalent binding interfaces. Entanglement is a recurring structural motif in nature, found in plant proteins,^[^ ^19^ ^]^ bacterial DNA,^[^ ^20^ ^]^ viral RNA,^[^ ^21^ ^]^ and bacteriophage capsids.^[^ ^22^ ^]^ Artificial entanglements have also been introduced in protein‐based therapeutics to enhance their affinity and proteolytic resistance.^[^ ^23^ ^]^ Yet, the potential of MIMs in biotechnologies remains largely untapped,^[^ ^24^, ^25^ ^]^ and only a few studies investigated polyrotaxanes for plasmid DNA^[^ ^26^, ^27^ ^]^ and RNA^[^ ^28^, ^29^ ^]^ delivery.

Here, we report the dynamic covalent synthesis of peptide‐based [2]catenanes presenting multiple cationic residues in a spatially controlled manner. We demonstrate that these interlocked structures exhibit enhanced proteolytic stability, effectively complex small‐interfering RNA (siRNA) in aqueous media, and improve its resistance to nuclease degradation.

## 2 Results and Discussion

### 2.1 Design and Synthesis of the [2]Catenanes

**Figure 1:**
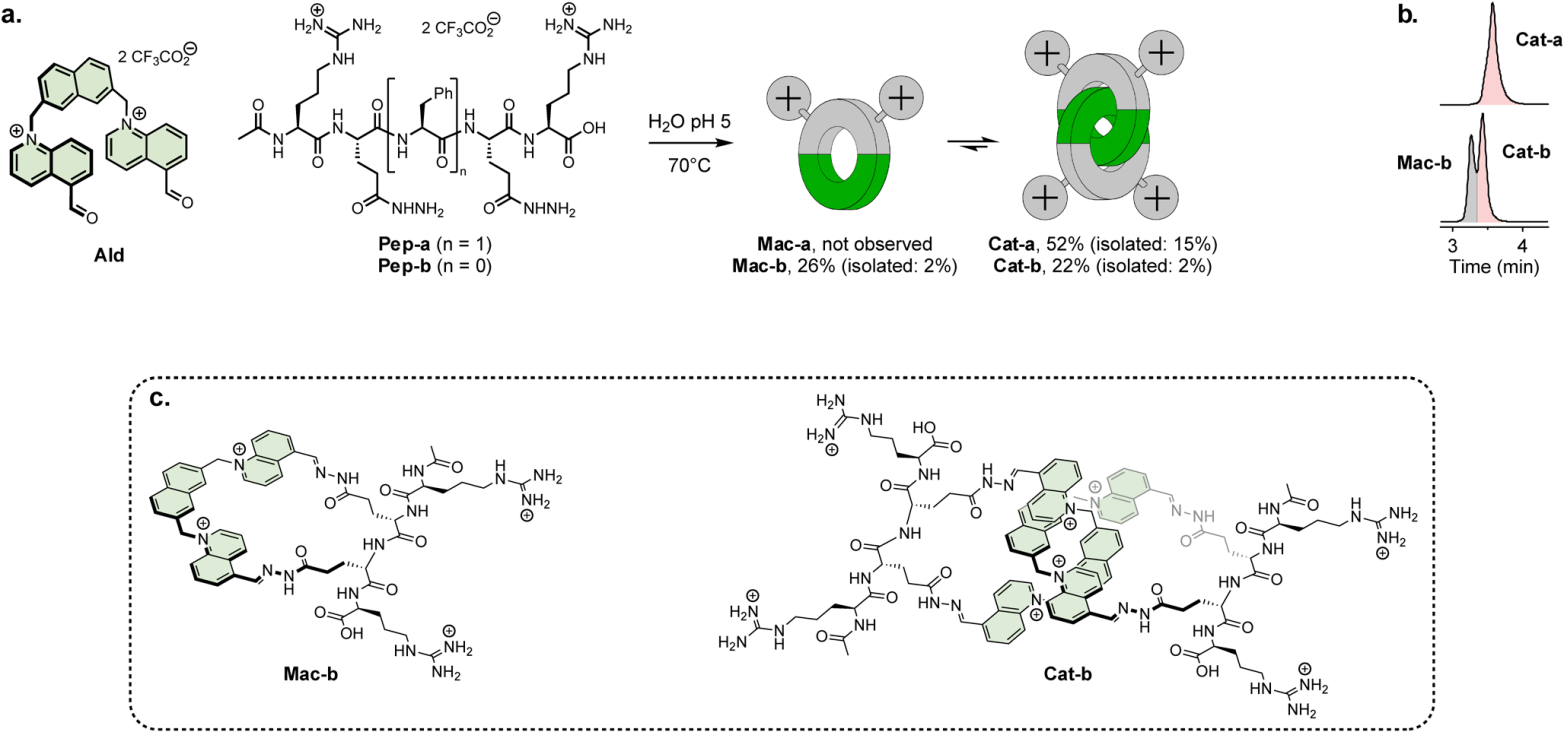
a) Dynamic covalent synthesis of macrocycle and [2]catenanes bearing *l*‐arginine residues. Conversion and isolated yields (in brackets) are given for each product. b) HPLC chromatograms of the reaction mixtures. c) Chemical structure of **Mac‐b** and **Cat‐b**.

The Cougnon group previously demonstrated that the reversible condensation of bisaldehyde **Ald** with flexible bishydrazydes in water yields [2]catenanes to minimize the hydrophobic surface area exposed to the solvent.^[^ ^30^ ^]^ This dynamic covalent methodology^[^ ^31^, ^32^, ^33^, ^34^, ^35^ ^]^ is simple, versatile, and extendable to more intricate topologies, including trefoil knots.^[^ ^36^, ^37^ ^]^ Building on this work, we hypothesized that appropriately designed bishydrazides could be used to introduce functional groups capable of interacting with nucleic acids (Figure 1a). Accordingly, we designed peptide‐based bishydrazide building blocks (**Pep‐a** and **Pep‐b**) bearing **l**‐arginine residues, which are well‐known to engage in salt‐bridge interaction with nucleic acids.^[^ ^8^, ^38^, ^39^ ^]^ An *l*‐phenylalanine residue was also incorporated in building block **Pep‐a** to probe the role of hydrophobic amino acids on nucleic acid binding. The choice of peptide‐based building blocks was driven by their water solubility, ease of synthesis, and modularity. Indeed, solid‐phase peptide synthesis (SPPS) allows for rapid preparation using commercially available Fmoc‐protected amino acids. Second, the versatility of SPPS enables variations in the peptide sequence, playing, for instance, with basic amino acids of different pKa (His, Lys, Arg) since the protonation degree is known to affect both nucleic acid complexation and cell uptake. Finally, amino acids and peptides are well‐established scaffolds for MIMs,^[^ ^40^ ^]^ particularly in the context of dynamic covalent chemistry.^[^ ^41^, ^42^, ^43^, ^44^, ^45^ ^]^ Given that acylhydrazone bonds are compatible with the presence of RNA,^[^ ^46^, ^47^, ^48^, ^49^, ^50^ ^]^ this approach represents an alternative to traditional metal coordination approaches used to access cationic clusters^[^ ^51^, ^52^ ^]^ and MIMs.^[^ ^31^, ^41^ ^]^

### 2.2 Synthesis

Bishydrazides **Pep‐a** and **Pep‐b** were synthesized via manual SPPS, using a modified *l*‐glutamic acid derivative functionalized with an acylhydrazide group, Fmoc‐*l*‐Glu[NHNH(Cl‐Trt)]‐OH.^[^ ^47^ ^]^ The peptides were first cleaved from the resin under mild conditions to preserve the arginine protecting group and facilitate purification by reverse‐phase high‐performance liquid chromatography (HPLC), followed by full deprotection and precipitation in cold diethyl ether, which afforded the desired products. The detailed synthetic protocols are described in the Supporting Information.

The corresponding cationic macrocycle and [2]catenanes were obtained by reacting **Ald** with either **Pep‐a** or **Pep‐b**. The building blocks were first dissolved in a 1:1 molar ratio (20 mM total) in Milli‐Q water. As the peptide‐based building blocks were used in their trifluoroacetic acid salt form, the solution pH was naturally around 5, without the need for adjustment. This mildly acidic pH is optimal for the formation and reversible exchange of acylhydrazone linkages. The solution was then stirred at 70 °C overnight. Although a significant amount of precipitate formed during the reaction, likely due to oligomerization, the crude mixtures obtained after filtration were remarkably simple (Figure 1b). In the case of **Pep‐a**, the reaction predominantly yielded the expected [2]catenane (**Cat‐a**) with a conversion of 52%, as determined by HPLC peak integration using hydroquinone as an internal standard. In contrast, the reaction of **Ald** with **Pep‐b** produced a mixture of [2]catenane **Cat‐b** and the intermediate [1 + 1] macrocycle **Mac‐b**, with conversions of 26% and 22%, respectively.

**Figure 2:**
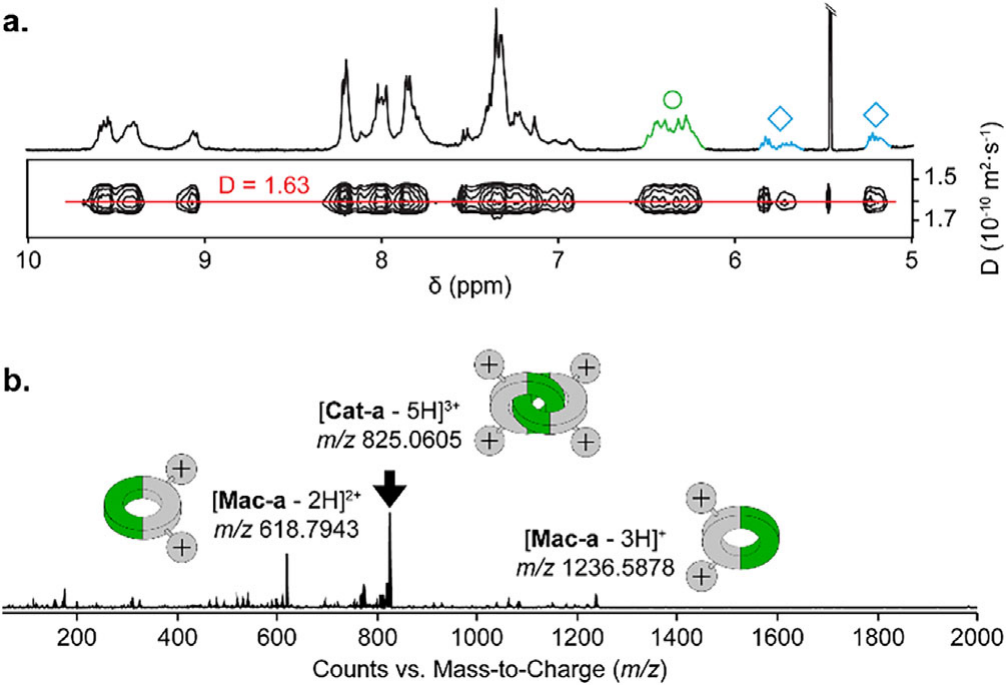
a) ^1^H and DOSY NMR characterization and b) MS/MS fragmentation (precursor ion: *m/z* 825, collision energy voltage: 20 V) of **Cat‐a**.

**Cat‐a**, **Cat‐b**, and **Mac‐b** were isolated by preparative reverse‐phase HPLC in yields of 15%, 2%, and 2%, respectively. The lower yields of **Cat‐b** and **Mac‐b** were attributed to their similar retention times, which hindered efficient separation. The products were characterized by nuclear magnetic resonance (NMR) and electrospray ionization mass spectrometry (ESI‐MS). Representative characterization data for **Cat‐a** are shown in Figure 2. Additional characterization for **Cat‐b** and **Mac‐b** is provided in the Supporting Information (Figures S14‐S18).

Due to the directionality of the peptide‐based building block, **Cat‐a** can exist as a mixture of two diastereomers,^[^ ^30^ ^]^ which may not be separable by chromatography and can adopt multiple conformations in slow exchange. As a result, the ^1^H NMR spectrum of **Cat‐a** in D₂O (Figure 2a) is complex but exhibits all the characteristic features of an interlocked structure.^[^ ^37^ ^]^ Due to aromatic stacking interactions, the quinolinium proton signals were significantly upfield‐shifted, and some signals (labeled with an empty diamond, ♢) even appeared below 6 ppm. In addition, the multiplicity of (‐CH₂‐) protons (labeled with an empty circle, °) was suggestive of diastereotopic environments. A single diffusion coefficient across all resonances further confirmed that these signals correspond to isomers of the same [2]catenane. Finally, tandem MS/MS analysis supported the interlocked nature of **Cat‐a**, revealing a fragmentation pattern consistent with a [2]catenane composed of two interlocked [1 + 1] macrocycles (Figure 2b).^[^ ^37^ ^]^

### 2.3 Resistance to Proteases

**Figure 3:**
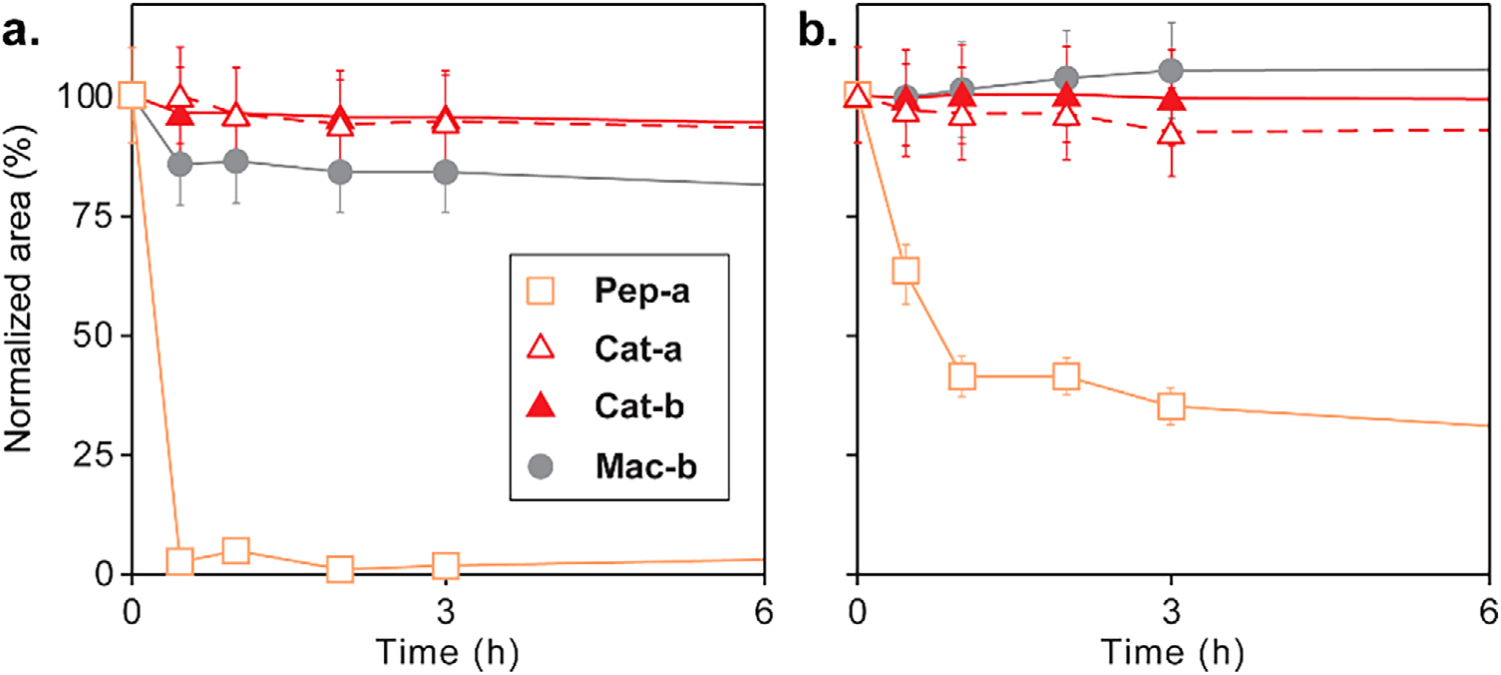
a) Trypsin and b) Proteinase K degradation of **Pep‐a**, **Cat‐a**, **Cat‐b**, and **Mac‐b**. Degradation of these compounds (2 mM) was assessed in the presence of trypsin or proteinase K (1 µg/µL) in 50 mM ammonium bicarbonate buffer (pH 8.4) at 35 °C. Reactions were monitored over time by collecting aliquots and analyzing them by HPLC. Degradation kinetics were determined by plotting normalized HPLC peak areas versus time. A standard error of 10% is shown to guide the eye.

The proteolytic stability of these compounds was monitored by HPLC after incubation at 37 °C in the presence of either trypsin or proteinase K, which cleave peptides at the carboxyl side of arginine and phenylalanine residues, respectively (Figures 3 and S19‐S22).^[^ ^53^ ^]^ Relative quantification by measuring peak area variations, normalized to the initial t_0_, reveals that **Pep‐a** was fully degraded after just 1 hour with trypsin and within a few hours with proteinase K. On the other hand, **Cat‐a** remained stable for at least 24 hours under both conditions. **Cat‐b** and **Mac‐b** also resisted proteolytic degradation, although macrocycle **Mac‐b** showed slightly reduced stability compared to its [2]catenane counterpart upon prolonged trypsin exposure. These results suggest that both macrocyclization and mechanical bond formation contribute to enhancing the stability of the peptide assemblies.

### 2.4 Complexation of Small‐Interfering RNA

We used an electrophoretic gel retardation assay^[^ ^47^, ^48^, ^49^ ^]^ to evaluate the ability of our compounds to complex a commercially available control siRNA (siCtrl), with the following sequences: 5′‐CGUACGCGGAAUACUUCGAdTdT‐3′ (sense strand) and 5′‐UCGAAGUAUUCCGCGUACGdTdT‐3′ (antisense strand). To determine the minimum concentration required for effective complexation, varying concentrations of each compound were incubated with siRNA in a saline phosphate buffer (150 mM NaCl, 25 mM phosphate, pH 7.2). Complexation efficiency was assessed by adjusting the nitrogen‐to‐phosphate (N/P) ratio, which represents the molar ratio of positively charged nitrogens from our compounds to negatively charged phosphates from siRNA. The N/P ratio was systematically varied from 1 to 100.

**Figure 4:**
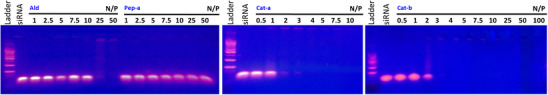
Gel electrophoresis assay showing improved complexation of siRNA by **Cat‐a**, compared to **Ald** and **Pep‐a** alone and **Cat‐b**. The compounds were incubated with a fixed concentration of siRNA (1.9 µM) in PBS buffer (25 mM phosphate, 150 mM NaCl, pH 7.2) to achieve the desired N/P ratios. Following a 30‐minute incubation at room temperature.

The samples were incubated for 30 minutes at room temperature and analyzed by agarose gel electrophoresis, where the fluorescent dye GelRed, which selectively binds noncomplexed siRNA, reveals the presence of free siRNA (Figure 4). As controls, we included a DNA ladder (100 bp increments from 100 to 1,000 bp) and free siRNA at the same concentration as in the test samples.

During electrophoresis, nucleic acids separate based on size and charge, with smaller and more negatively charged species migrating faster. Consequently, unbound siRNA migrates similarly to the free siRNA control, while siRNA complexed with cationic compounds exhibits reduced mobility.

The siRNA complexation ability of **Cat‐a** was first compared to that of its individual building blocks, **Pep‐a** and **Ald**. As expected, **Pep‐a** did not induce any siRNA complexation up to an N/P ratio of 50 (Figure 4). **Ald** showed complexation only at high N/P ratios (N/P > 25), likely due to the intercalation of the aromatic moieties between the base pairs of siRNA. In contrast, **Cat‐a** demonstrated markedly improved complexation ability, with complex formation observed at an N/P ratio as low as 2. **Cat‐b** exhibited similarly sharp complexation, with complete binding at N/P > 3 (Figure 4). These values compare very well with the efficacy of other cationic clusters to complex nucleic acids.^[^ ^7^, ^51^, ^52^ ^]^ This result indicates that the presence of a phenylalanine residue does not significantly affect the binding affinity. In contrast, both multivalency and structural preorganization are essential for efficient siRNA complexation. The enhanced binding observed with the [2]catenanes was therefore attributed to the precise three‐dimensional arrangement of its four *l*‐arginine residues. This interpretation was further supported by the observation that **Cat‐b** showed slightly better complexation efficiency than its macrocyclic counterpart **Mac‐b**, which displayed more gradual siRNA binding beginning at N/P > 7.5 (Figure S23). The difference in affinity, though modest, is notable given that the two compounds are structurally similar and form nanoparticles of comparable sizes upon complexation of siRNA, as determined by dynamic light scattering (238 ± 26 nm for **Cat‐b**; 244 ± 26 nm for **Mac‐b**, Figure S24).

### 2.5 Resistance to Nucleases

**Figure 5:**
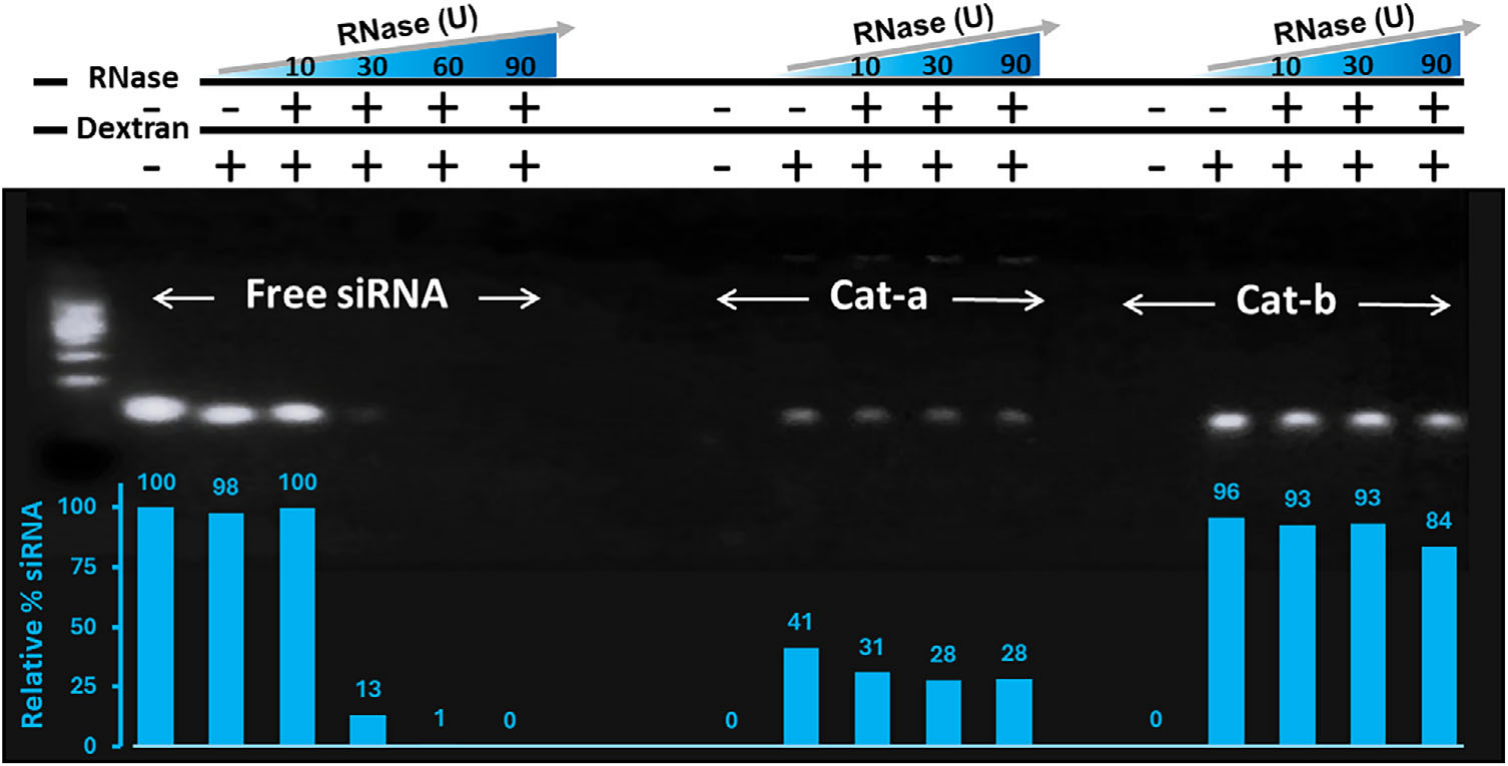
Gel electrophoresis assay of the enzymatic degradation by endonuclease RNase I of free siRNA and the siRNA complexes formed with **Cat‐a** and **Cat‐b** at N/P 10, with the histogram representing the relative % of siRNA determined by band quantification using ImageJ. Preformed siRNA complexes were incubated in phosphate buffer (20 mM, pH 7.2, without NaCl) at 37 °C for 1 hour in the presence of human pancreatic RNase I. The reaction was quenched by the addition of EDTA, followed by the addition of excess 8 kDa dextran to denature any remaining complexes. This mixture was further incubated for 1 hour at 37 °C prior to analysis.

The stability of the siRNA complexes against degradation by nucleases was assessed following a procedure recently reported.^[^ ^54^ ^]^ Preformed siRNA complexes were incubated in phosphate buffer (20 mM, without NaCl, pH 7.2) for 1 hour at 37 °C with varying concentrations of human pancreatic RNase I, an endonuclease degrading both single‐stranded and double‐stranded RNA. EDTA was added to quench the reaction, followed by incubation with an excess of 8 kDa dextran for 1 hour at 37 °C to denature the remaining complexes and release any intact siRNA. The samples were then analyzed by gel electrophoresis (Figure 5). Control experiments showed that treatment with 60 units of RNase I led to complete degradation of unprotected siRNA. In contrast, siRNA complexed with either **Cat‐a** or **Cat‐b** exhibited substantial, yet different, resistance to enzymatic degradation, with up to 84–96% intact siRNA released after harsh RNase I (90 units) treatment and dextran‐induced decomplexation.

## 3 Conclusion

In conclusion, we have demonstrated the dynamic covalent synthesis of water‐soluble cationic [2]catenanes capable of nucleic acid recognition. The synthesis was achieved through a simple process involving the condensation of an aromatic bisaldehyde with peptide‐based bishydrazides bearing *l*‐arginine residues. We showed that both macrocyclization and mechanical bond formation impart enhanced proteolytic stability to the peptide backbone. Gel electrophoresis assays confirmed that these [2]catenanes efficiently complex siRNA and, more importantly, confer resistance to nuclease degradation. The enhanced structural preorganization imparted by the formation of mechanical bonds is likely to become increasingly beneficial as the size and flexibility of the macrocyclic scaffold increases: while larger macrocycles tend to be conformationally dynamic, mechanically interlocked analogues can maintain a well‐defined preorganized structure, provided they possess a sufficient number of crossings.

Collectively, these results highlight the potential of MIMs as platforms for nucleic acid recognition and delivery. Such peptide‐based interlocked molecules are also appealing because their design is modular and allows for the incorporation of additional targeting or cell‐penetrating motifs to facilitate selective cellular uptake in therapeutic applications.

## Supporting Information

The authors have cited additional references within the Supporting Information.

## Conflict of Interest

The authors declare no conflict of interest.

## Supporting information



Supporting Information

## Data Availability

The data that support the findings of this study are available in the supplementary material of this article.
