# Supplementary material for: Effective RNA Complexation by [2]Catenanes Confers Enhanced Resistance to Enzymatic Degradation
Source: Chemistry. 2025 Jul 24;31(45):e01631. doi: 10.1002/chem.202501631 (PMC12351436; doi:10.1002/chem.202501631)
Supplement: Supplementary file 1 — Supporting Information [file CHEM-31-e01631-s001.pdf]

Supporting Information for:

Effective RNA Complexation by [2]Catenanes Confers Enhanced Resistance to Enzymatic Degradation

Dimitri Delcourt,[a]† José García Coll,[b]† Fabien B. L. Cougnon,[a]* and Sébastien Ulrich[b]*

a Department of Chemistry, Nanoscience Center, University of Jyväskylä, P.O. Box 35, FI-40014 JYU, Finland

b Institut des Biomolécules Max Mousseron (IBMM), Université de Montpellier, CNRS, ENSCM, Montpellier, France

† These authors contributed equally to this work

1 Materials and methods 2
2 Synthesis and characterization of the peptide building blocks 5
3 Synthesis and characterization of [2]catenanes and macrocycles 11
4 Protease Assays 18
5 Gel electrophoresis assays 22
6 Dynamic light scattering 23
7 References 24

1 Materials and methods

All reagents and solvents were purchased from commercial sources and used without further purification. Reagents for synthesis were purchased from BLD Pharma and Sigma Aldrich. Amino acids were purchased from IrisBiotech and BLD Pharma. **Ald**,[1] Fmoc-*L*-Glu[NHNH(Cl-Trt)]-OH,[2] and **ArgHyd**[3] were synthesized according to previously reported protocols. The sequence of the siRNA "siCtrl", which was purchased from Eurogentec (Serring, Belgium), is: 5'-CGUACGCGGAAUACUUCGAdTdT-3' (sense strand) and 5'-UCGAAGUAUUCCGCGUACGdTdT-3' (anti-sense strand). Trypsin was purchased from Sigma Aldrich, proteinase K, dextran sulfate sodium and Ambion™ RNase I were purchased from Thermo Fischer Scientific.

Solid Phase Peptide Synthesis (SPPS). All peptide syntheses were performed on a 2-chlorotrityl chloride resin (loading 1.60 mmol/g) following a Fmoc strategy. These syntheses were carried out manually at room temperature on a scale of 0.37 mmol.
Briefly, the loading started with the 2-chlorotrityl chloride resin (1 eq.) being suspended in a solution of DMSO/NMP (8/2), before adding DIEA (4 eq.), then the suspension was gently stirred. Next, Fmoc-*L*-Arg(Pbf)-OH (1 eq.) was added in one portion, and the reaction was allowed to react for 72 h. Finally, resin was filtered and capped with MeOH, followed by thorough wash with DMF. A resin loading of 0.27 mmol/g was then determined by the Fmoc absorbance measurement, as previously described.[2, 4]
The following elongation conditions were then used for the peptide synthesis:

- Resin deprotection (Fmoc removal): piperidine/DMF (2/8) at r.t. for 2 min (twice).
- Coupling conditions: Fmoc-AA-OH 0.6 M in DMF (5 eq.), HATU 0.2 M in DMF (5 eq.), DIEA (10 eq.), stirred for 10 min. Double coupling was used except for the modified amino acid Fmoc-*L*-Glu[NHNH(Cl-Trt)]-OH.
- Fmoc deprotection conditions: piperidine/DMF (2/8) for 5 min (twice).
- N-terminal acetylation: Ac_2O (150 eq.)/DCM 1/1 (v/v) for 5 min (twice).
- Cleavage conditions:
  a) Mild cleavage conditions: TFA/DCM (1/99) for 5 min, 4 times, then MeOH/Pyridine (8/2). Mild cleavage conditions were used to cleave the peptide without deprotecting the arginine side chain Pbf groups in order to facilitate purification by reverse-phase HPLC. Note that the Cl-Trt protecting groups are deprotected under these conditions.
  b) Final deprotection conditions (after mild cleavage): TFA/TIS/H_2O (95/2.5/2.5) solution at 3 mM concentration of protected peptide for 3 hours at room temperature.
- Peptide recovery after cleavage: filtration of the resin, concentrated *in vacuo*, precipitated with ice cold Et_2O and supernatant removal.
- Reverse-phase HPLC purification.
- Crude product is freeze-dried to obtain the final peptides as light powders.

Determination of peptide stock solution concentration. The final peptides were all titrated by ^1H NMR (in D_2O) using *tert*-butanol as an internal reference to determine the exact concentration. For this titration, the compound was solubilized in D_2O (final concentration around 30 mM) and *tert*-butanol was added (50 µL of a 30 mM solution in D_2O) in the NMR tube (total volume of 500 µL). ^1H NMR spectrum was recorded, and the relative peak

integration was used to calculate the exact concentration of peptide. Indicated yields are therefore calculated from the titration value.

General protocol for the synthesis of catenanes and macrocycles. Building blocks **Ald** (10 mM) and **Pep** (10 mM) were dissolved in Milli-Q water (10 mL). The measured pH was around 5 (without adjustment). The solution was heated overnight at 70 ºC to reach equilibrium, and the reaction was monitored by UHPLC-MS. After filtration, the resulting catenanes and macrocyles were isolated as trifluoroacetate salts by semi-preparative reverse-phase HPLC. Each fraction was frozen as soon as it was collected to avoid reorganisation of the dynamic acylhydrazone bond. The combined fractions were lyophilized, yielding each product as a fluffy yellow powder.

NMR. ^{1}H NMR spectra were recorded at 400 MHz on Bruker Avance 400 instruments and on a Bruker Avance III 500 MHz spectrometer equipped with a 5 mm DCH ^{13}C-^{1}H/D helium-cooled cryogenic probe in deuterated solvents. Peaks were referenced in ppm with respect to the residual solvent peak. Data are reported as follows: chemical shift (δ in ppm), multiplicity (s for singlet, d for doublet, t for triplet, m for multiplet), coupling constant (*J* in Hertz), and integration.

Analytical HPLC analyses. Analytical reverse-phase HPLC (RP-HPLC) analyses were performed on a Thermo Scientific™ - UltiMate™ 3000 UHPLC system equipped with a Thermo Scientific™ Hypersil GOLD™ aQ C18 Polar Endcapped HPLC Column 25302-052130, (1.9 µm, 2.1 x 50 mm) column and a Thermo Scientific™ Dionex™ UltiMate™ DAD 3000 detector. Retention times (t_R) are given in minutes.

Preparative HPLC. Peptide building blocks were purified on a Gilson® PLC 2250 Purification System equipped with a UV-Vis Glison® DAD detector and using a gradient of solution A (99.9% water, 0.1% trifluoroacetic acid) and solution B (99.9% acetonitrile, 0.1% trifluoroacetic acid). The purification system was equipped with a WATERS™ XSelect™ CSH C18 OBD preparative-scale column 186005493, (130 Å, 5 µm, 30 nm x 250 nm). [2]Catenanes and [1+1] macrocycle were purified by HPLC Shimadzu LC-8A system equipped with a Shimadzu array detector SPD-M20A using a Gemini 10 µm C18 column 110 Å, 100 x 21.20 mm 10 micron from Phenomenex.

UHPLC-MS. UHPLC-MS analyses were performed on an Agilent 6530-QTOF mass spectrometer (ionisation mode: ESI+) equipped with an Agilent 1290 UHPLC inlet, UV detector and autosampler. MS-MS fragmentation was peformed on the same instrument. Retention times (t_R) are given in minutes.

HR-ESI-MS. Analyses were carried out at the *Laboratoire de Mesures Physiques, IBMM, Université de Montpellier* using Micromass QTOF instruments.

Gel retardation assay. In PBS buffer (25 mM, 150 mM NaCl, pH 7.2), a fixed concentration (1.9 µM) of siCtrl was mixed with the appropriate amounts of monomer, macrocycle or catenane to reach the desired N/P ratios in a final volume of 20 µL. After 30 min of incubation at room temperature, 5 µL of blue 6X loading dye (Fisher Scientific) was added to the mixture. Electrophoresis was carried out on a 2 % w/v agarose gel mixed with GelRed^{TM} nucleic acid gel stain (Interchim, France) in 1X TBE buffer (90 mM Tris-borate/2 mM EDTA, pH 8.2). The gel was run in 0.5X TBE at 100 V for 20 min. A 100 bp DNA ladder from Sigma-Aldrich (Saint-Quentin-Fallavier, S4 France) was used as a reference for the gel. The GelRed-stained

siRNA was visualized using a TFX-20 M model-UV transilluminator (Vilber Lourmat, Marne-la-Vallée, France) and gel photographs were obtained with a smartphone camera.

Dynamic light scattering. Measurements were performed using Zetasizer Nano-ZS instrument (Malvern, United Kingdom) with transparent ZEN0040 disposable micro-cuvette (40 µL) at 25 °C. Samples were prepared as described in the gel retardation assay at N/P 10 and diluted to 50 nM concentration of siRNA with the same buffer prior to analysis.

RNase assay. The procedure was adapted from the litterature.[5] In phosphate buffer (20 mM, without NaCl, pH 7.2), a fixed concentration (2.7 µM) of siCtrl was mixed with the appropriate amount of compound (1 µL at 2 mM in H_2O) to achieve N/P 10 in a final volume of 14 µL. After 1 hour of incubation at 37 ºC, different units of RNase were added (10, 30, 60 and 90 units or the equivalent for 4, 12, 24, 36 units/µg of siRNA) in a final volume of 15 µL. After 1 hour of incubation at 37 ºC, RNase was quenched by cooling down the samples to 0 ºC and adding EDTA (1 µL at 500 mM, 5 min incubation). Dextran (8 kDa) was then added (8 µL at 4.4 mM) to each sample for a N/P of 20 and a final volume of 24 µL (final siCtrl concentration 1.6 µM). After 1 hour of incubation time at 37 ºC, 6 µL of freshly prepared loading buffer (10 mM Tris-HCl pH 7.6 40%, 60% glycerol) was added to the mixture. Electrophoresis was carried out as previously described.

Protease assay. The procedure was adapted from the litterature.[6] For each assay, 1 µL of a solution of product is added to 199 µL of a solution of protease. Stock solution of **Pep-a** was prepared at 10 mM concentration in distilled water. Stock solutions of **Cat-a**, **Mac-b** and **Cat-b** were prepared at 2 mM concentration in distilled water. Stock solutions of trypsin and proteinase K were prepared at 1 µg/µL concentration in a 50 mM ammonium bicarbonate buffer at pH 8.4. All degradations assays were performed in Eppendorf tubes held at 35 °C with orbital mixing (300 rpm). The degradation after different time points (0.5, 1, 2, 3 and 24 h) was monitored by direct injection of 6 µL aliquots in HPLC where the acidic eluent (0.1% TFA) quenches the reaction. Absorbance was recorded at 214 nm (for **Pep-a**) and 254 nm (for **Cat-a**, **Mac-b** and **Cat-b**). Control experiments were carried out with the same protocol.

List of abbreviations. AA: Amino acid. Ac: Acetyl protecting group. Ac_2O: Acetic anhydride. bp: Base pair. calc.: calculated. ClTrt: 2-Chlorotrityl protecting group. DCM: Dichloromethane. DIEA: *N,N*-diisopropylethylamine. DMF: *N,N*-Dimethylformamide. DMSO: Dimethyl sulfoxide. DNA: Deoxyribonucleic acid. DOSY: Diffusion ordered spectroscopy. EDTA: Ethylenediaminetetraacetic acid. eq.: Equivalent. ESI: Electrospray ionization. Et_2O: Diethyl ether. Fmoc: Fluorenylmethoxycarbonyl protecting group. HATU: 1-[Bis(dimethylamino)methylene]-1H-1,2,3-triazolo[4,5-b]pyridinium 3-oxide hexafluorophosphate. HPLC: High-performance liquid chromatography. HR-MS: High-resolution mass spectrometry. UHPLC-MS: Ultra-high-performance liquid chromatography-mass spectrometry. *m/z*: mass-to-charge ratio. mAU: Milli-absorbance unit. MeOH: Methanol. MS/MS: Tandem mass spectrometry. NMP: *N*-Methyl-2-pyrrolidone. NMR: Nuclear magnetic resonance. Pbf: 2,2,4,6,7-pentamethyldihydrobenzofuran-5-sulfonyl protecting group. PBS: Phosphate-buffered saline. PDI: Polydispersity index. Ph: Phenyl group. ppm: Parts per million. Prot: Protected. QTOF: Quadrupole time-of-flight. RNA: Ribonucleic acid r.t.: Room temperature. siRNA: Small-interfering RNA. SPPS: Solid-phase peptide synthesis. TBE: Tris-Borate-EDTA. *t*-BuOH: *Tert*-Butyl alcohol. TFA: Trifluoroacetic acid. TIS: Triisopropyl silane. Tris: 2-amino-2-hydroxyméthylpropane-1,3-diol. UHPLC: Ultra-high-performance liquid chromatography. UV-vis: Ultraviolet-visible.

2 Synthesis and characterization of the peptide building blocks

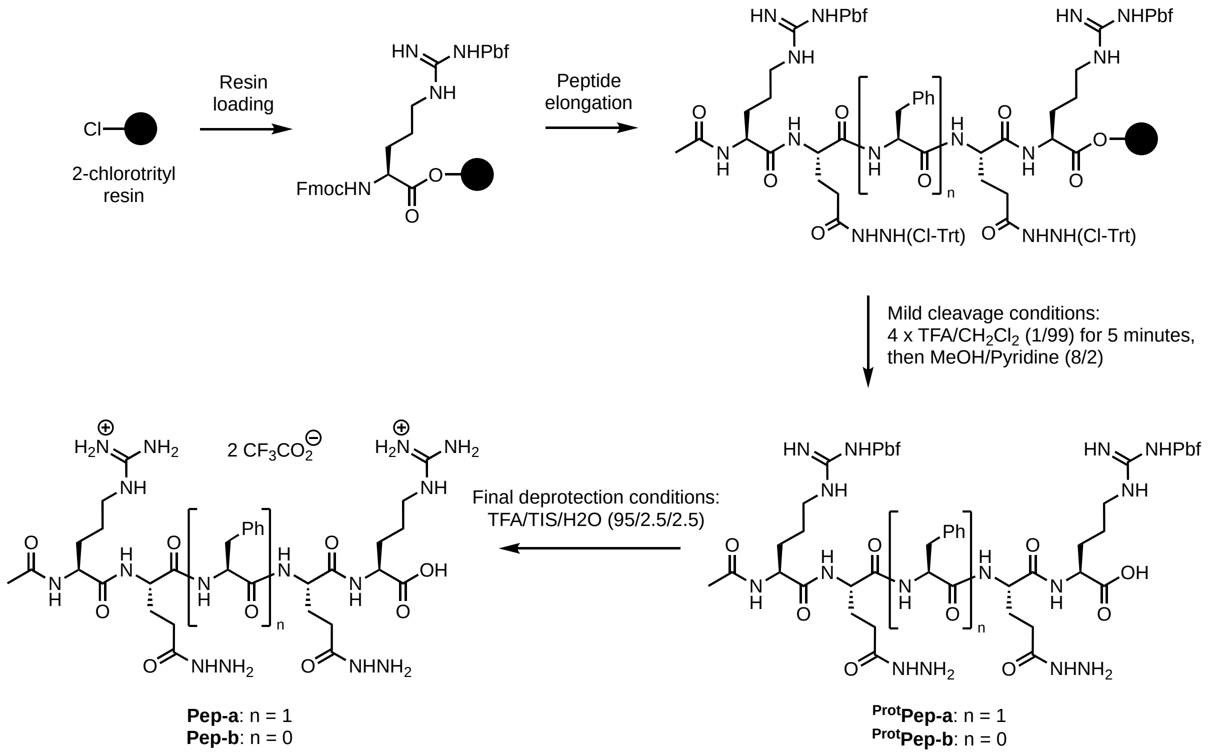

Scheme S1. Synthetic route for the preparation of **Pep-a** and **Pep-b**.

^{Prot}Pep-a was synthesized according to the general SPPS procedure using Fmoc-*L*-Arg(Pbf)-OH, Fmoc-*L*-Phe-OH and Fmoc-*L*-Glu[NHNH(Cl-Trt)]-OH. The desired product was obtained after HPLC preparative purification. Eluents: solution A (99.9% H_2O, 0.1 % TFA), solution B (99.9% acetonitrile, 0.1% TFA). Gradient: 0 min, 30% B; 5 min, 30% B; 45 min, 100% B. Flow rate: 45 mL/min. Yield: 145 mg (30%). HR-ESI-MS *m/z* calc. for $[C_{59}H_{87}N_{15}O_{15}S_2+H]^+$ 1310.6020, found 1310.5980; $[C_{59}H_{87}N_{15}O_{15}S_2+2H]^{2+}$ 655.8047, found 655.8040; $[C_{59}H_{87}N_{15}O_{15}S_2+3H]^{3+}$ 437.5389, found 437.5378.

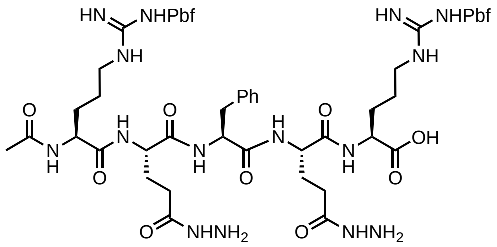

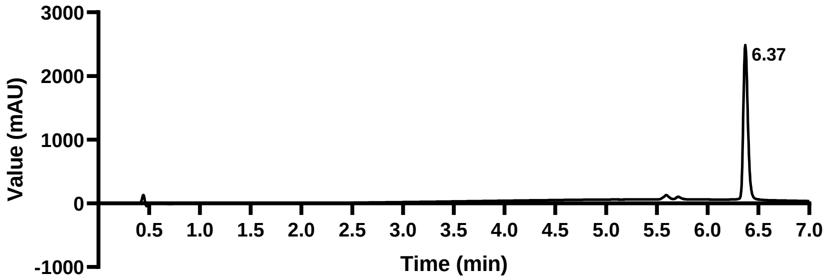

Figure S1. HPLC chromatogram of **^{Prot}Pep-a** (t_R: 6.37 min). Eluents: solution A (99.9% water, 0.1 % trifluoroacetic acid), solution B (99.9% acetonitrile, 0.1 % trifluoroacetic acid). Gradient: 5% to 100% B in 5 min, then up to 10 min at 100% B. Flow rate: 0.5 mL/min.

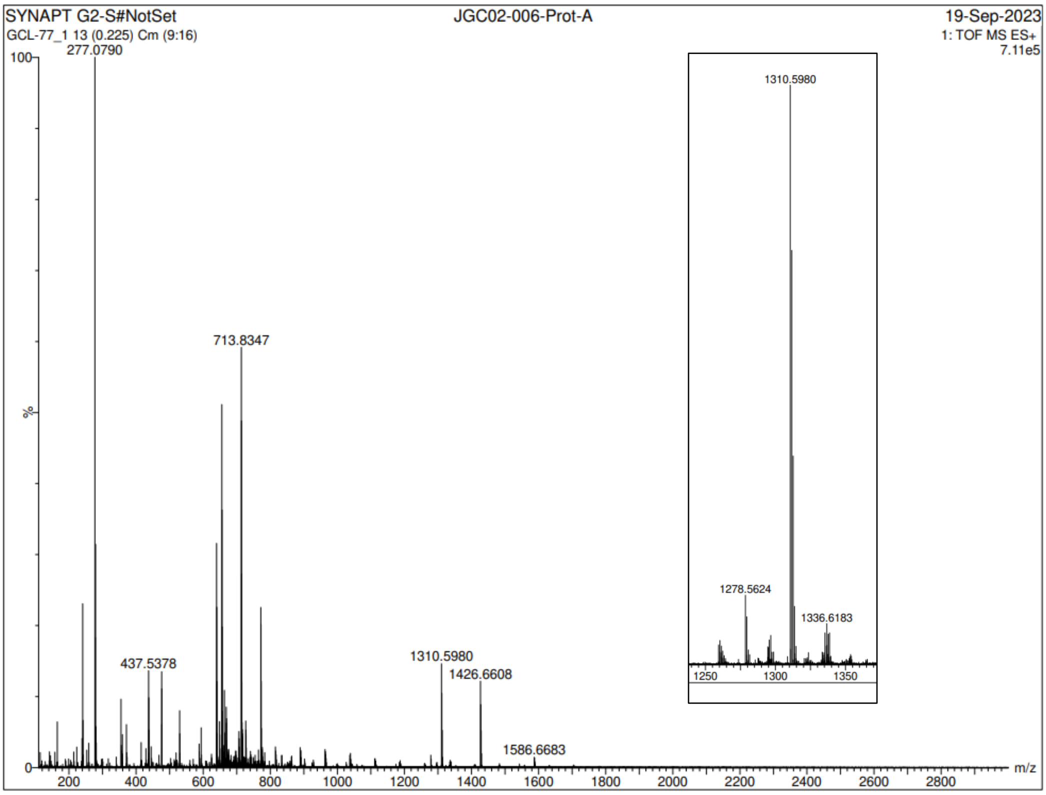

Figure S2. ESI-MS spectrum of **^{Prot}Pep-a**.

Pep-a was obtained by deprotecting compound **ProtPep-a** according to the general SPPS procedure. Yield: 66 mg (75%) as a white solid. HR-ESI-MS *m/z* calc. for $[C_{33}H_{55}N_{15}O_9+H]^+$ 806.4380, found 806.4369; $[C_{33}H_{55}N_{15}O_9+2H]^{2+}$ 403.7226, found 403.7228. ^{1}**H NMR** (D_2O, 298 K) δ_H: 7.38-7.21 (m, 5H, Ar_F), 4.68 - 4.59 (4.45 - 4.32, m, 1H, α_F), 4.41 - 4.27 (m, 3H, $H\alpha_{E*}$ + $H\alpha'_R$), 4.24 - 4.15 (m, 1H, $H\alpha_R$) 3.29 - 3.09 (m, 5H, $H\delta_R$ + $H\beta_F$), 3.05 – 2.95 (m, 1H, $H\delta_R$) 2.50 - 1.50 (m, 16H, $H\beta_R$ + $H\beta_{E*}$ + $H\gamma_R$ + $H\gamma_{E*}$), 2.05 (s, 3H, CH_3).

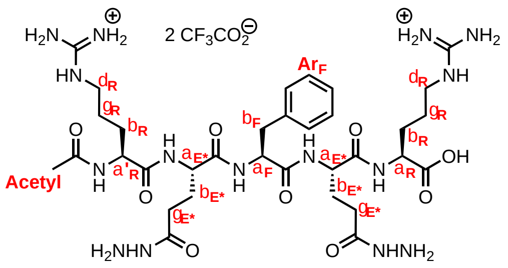

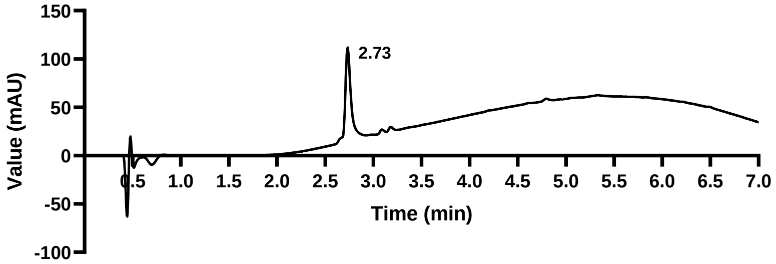

Figure S3. HPLC chromatogram of **Pep-a** (t_R: 2.73 min). Eluents: solution A (99.9% water, 0.1 % trifluoroacetic acid), solution B (99.9% acetonitrile, 0.1 % trifluoroacetic acid). Gradient: 0% to 100% B in 5 min, then up to 10 min at 100% B. Flow rate: 0.5 mL/min.

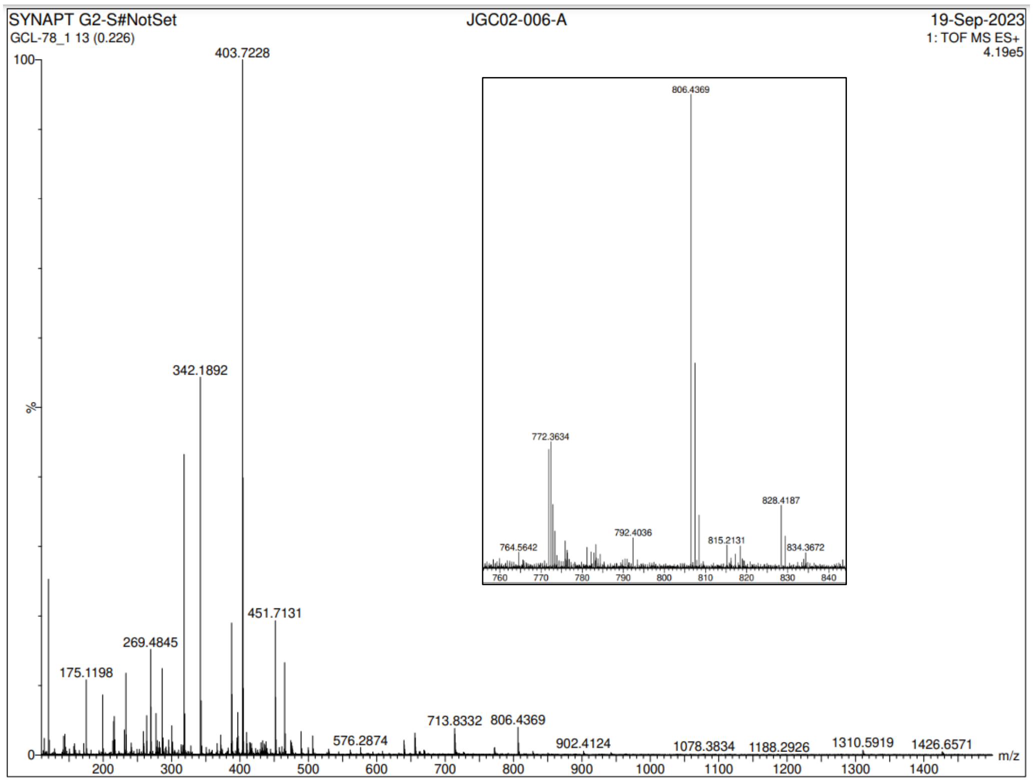

Figure S4. HR-ESI-MS Spectrum of **Pep-a**.

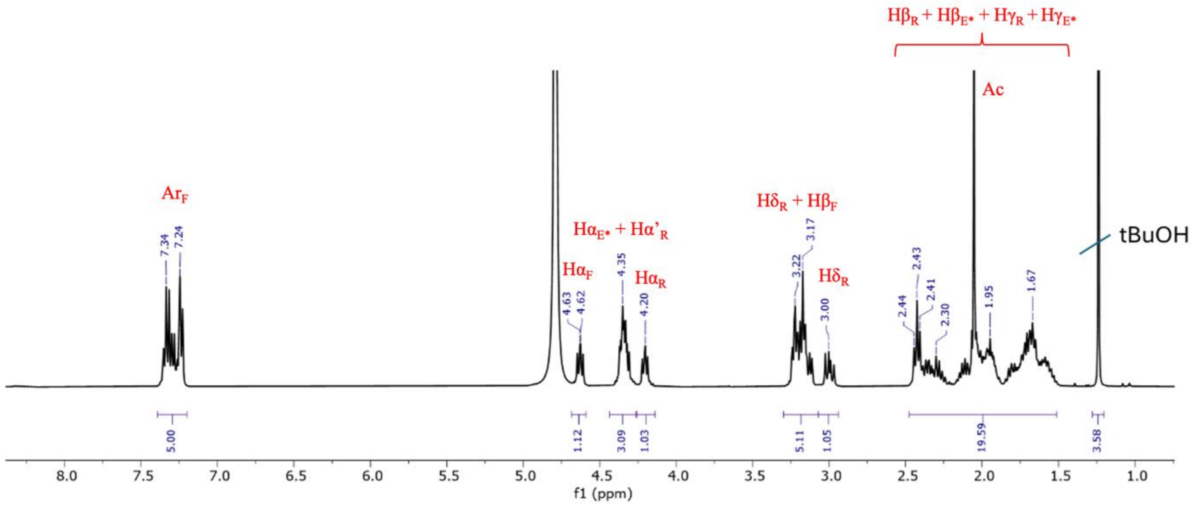

Figure S5. ^{1}H NMR spectrum of **Pep-a** (D_2O, 298 K, internal standard: *t*BuOH).

ProtPep-b was synthesized according to the general SPPS procedure using Fmoc-*L*-Arg(Pbf)-OH and Fmoc-*L*-Glu[NHNH(Cl-Trt)]-OH. Compound **ProtPep-b** was obtained after preparative purification. Eluents: solution A (99.9% H_2O, 0.1 % TFA), solution B (99.9% acetonitrile, 0.1 % TFA). Gradient: 0 min, 30% B; 5 min, 30% B; 45 min, 100% B. Flow rate: 45 mL/min. Yield: 105 mg (25%). ESI-MS *m/z* calc. for $[C_{50}H_{78}N_{14}O_{14}S_2+H]^+$ 1163.53, found 1163.45; $[C_{50}H_{78}N_{14}O_{14}S_2+2H]^{2+}$ 582.27, found 582.50.

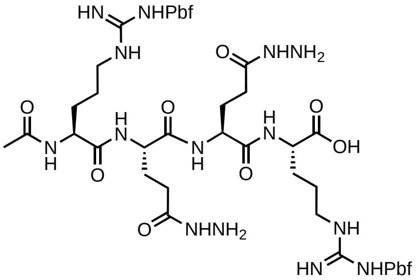

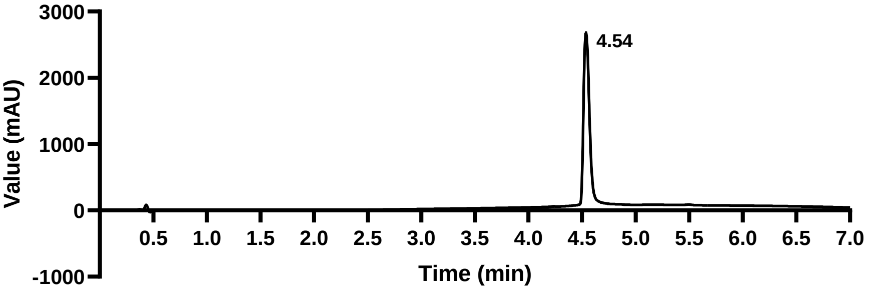

Figure S6. HPLC chromatogram of **ProtPep-b** (t_R: 4.54 min). Eluents: solution A (99.9% water, 0.1 % trifluoroacetic acid), solution B (99.9% acetonitrile, 0.1 % trifluoroacetic acid). Gradient: 5% to 100% B in 5 min, then up to 10 min at 100% B. Flow rate: 0.5 mL/min.

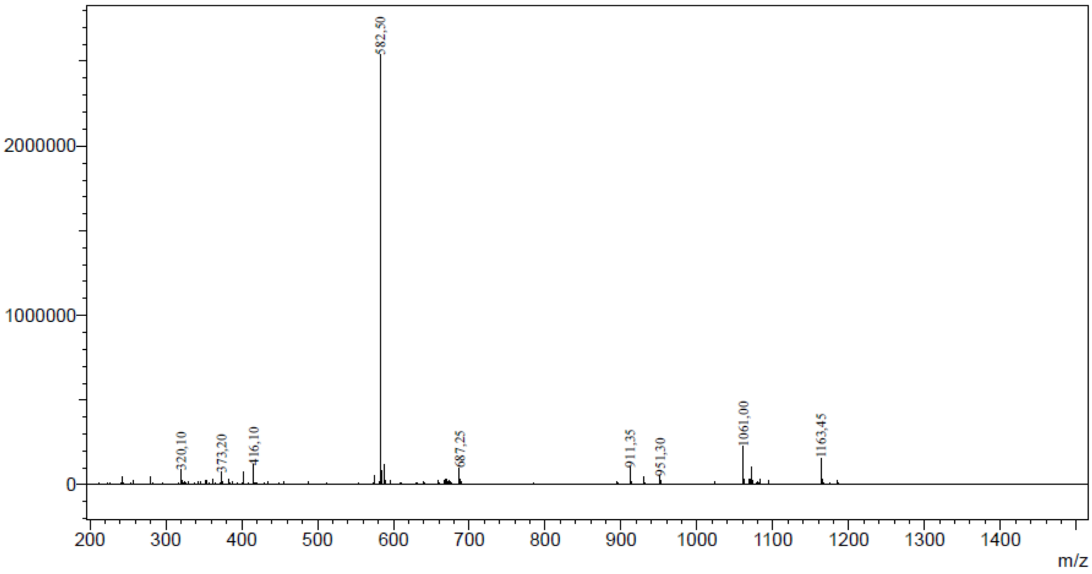

Figure S7. ESI-MS spectrum of **ProtPep-b**.

Pep-b was obtained by deprotecing compound **^{Prot}Pep-b** according to the general SPPS procedure. Yield: 45 mg (74%) as a white solid. The compound could not be characterized by HPLC as there was no retention on reverse phase. HR-ESI-MS *m/z* calc. for $[C_{24}H_{46}N_{14}O_8+H]^+$ 659.3696, found 659.3681; $[C_{24}H_{46}N_{14}O_8+2H]^{2+}$ 330.1884, found 330.1893. ^{1}H NMR (D_2O, 298 K) δ_H: 4.45 - 4.32 (m, 3H, $H\alpha_{E*}$ + $H\alpha'_R$), 4.32 - 4.23 (m, 1H, $H\alpha_R$) 3.28 - 3.15 (m, 4H, $H\delta_R$), 2.55 - 2.40 (m, 4H, $H\gamma_{E*}$), 2.23 - 1.72 (m, 8H, $H\beta_{E*}$ + $H\beta_R$), 2.04 (s, 3H, CH_3), 1.72 - 1.57 (m, 4H, $H\gamma_R$).

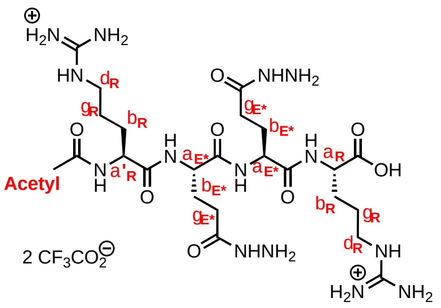

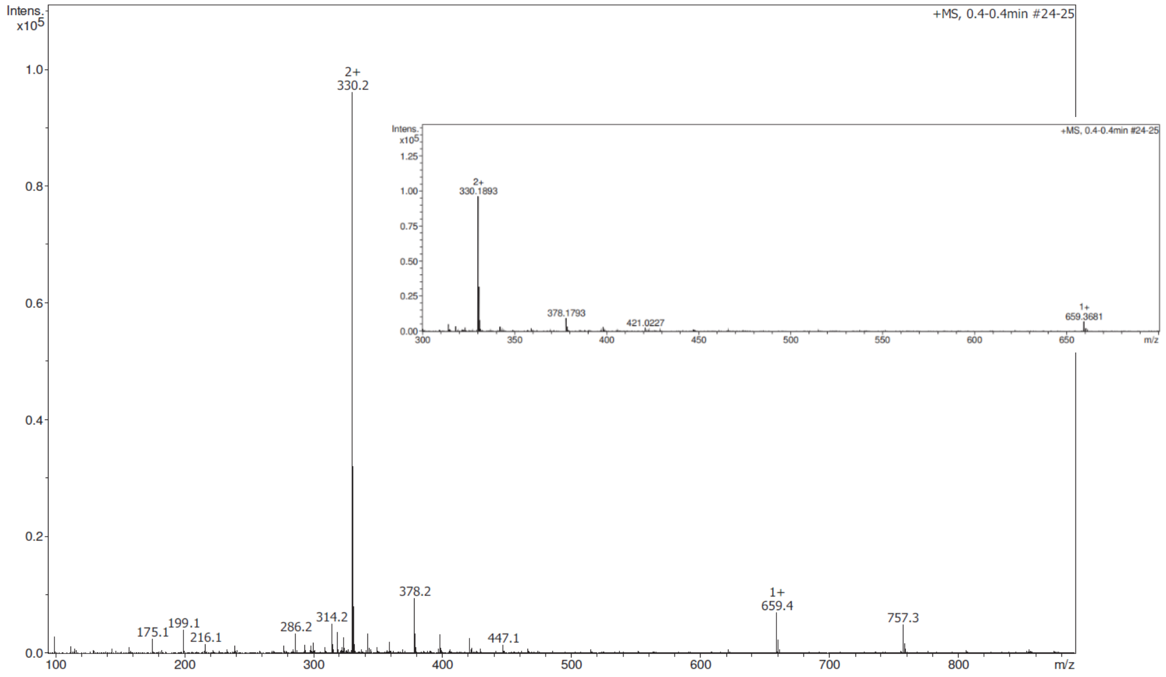

Figure S8. HR-ESI-MS spectrum of **Pep-b**.

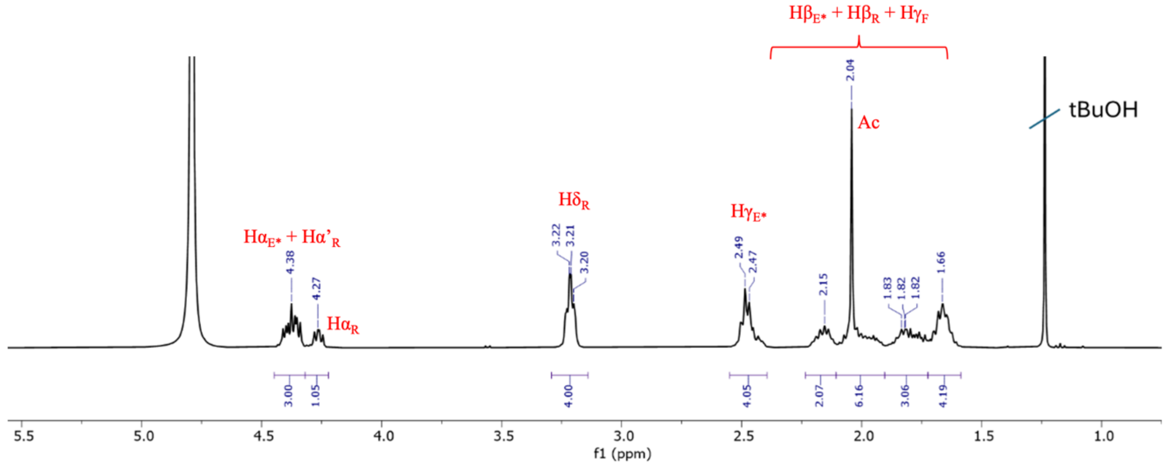

Figure S9. ^{1}H NMR spectrum of **Pep-b** (D_2O, 298 K, internal standard: *t*BuOH).

3 Synthesis and characterization of [2]catenanes and macrocycles

Cat-a. [2]catenane **Cat-a** was synthesized according to the general procedure and purified by preparative HPLC. Eluents: solution A (99.9% water, 0.1 % trifluoroacetic acid), solution B (99.9% acetonitrile, 0.1 % trifluoroacetic acid). Gradient: 0 min, 5% B; 1 min, 5% B; 15 min, 100% B. Flow rate: 12 mL/min. Yield: 6.95 mg (15%). HR-ESI-MS *m/z* calc. for $[C_{130}H_{154}N_{34}O_{18}\text{-}5H]^{3+}$ 825.0602, found 825.0616; $[C_{130}H_{154}N_{34}O_{18}\text{-}4H]^{2+}$ 619.0470, found 619.0482. ^{1}H NMR (D_2O, 298 K) δ_H: 9.93 – 8.78 (m, 8H, H_5+H_7), 8.29 – 7.65 (m, 4H, H_1[E/Z isomer]) + (m, 16H, H_2+H_3+H_4+H_6), 7.60 – 6.87 (m, 10H, Phe) + (m, H_9+H_{10}+H_{11}), 6.55 – 6.13 (m, H_8), 5.89 – 5.60 and 5.29 – 5.07 (m, upfield H_9+H_{10}+H_{11}), 5.45 (s, upfield H_8), 4.80 – 4.00 (m, Hα), 3.46 – 2.80 (m, 12H, $H\delta_R$+$H\beta_F$), 2.80 – 1.2 (m, 16H, $H\beta_R$ + $H\gamma_R$) + (m, 16H, $H\beta_E$ + $H\gamma_E$) + (m, 6H, H_{Acetyl}).

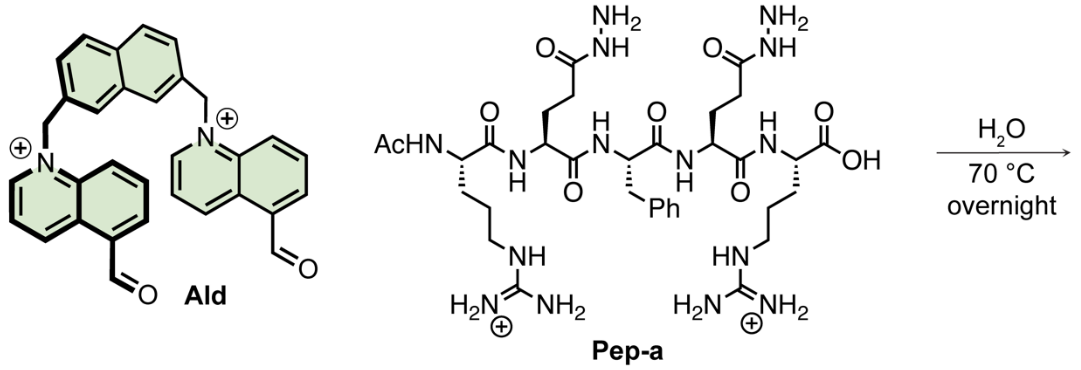

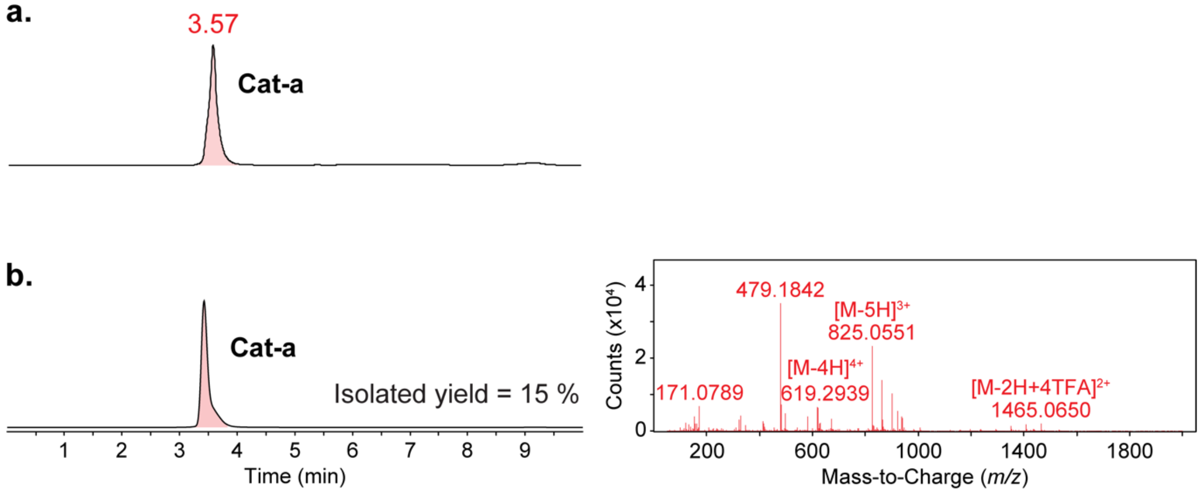

Figure S10. UHPLC-MS analysis of (a) the crude reaction mixture generated from **Ald** and **Pep-a**, and (b) isolated **Cat-a** (isolated yield: 15%), with the corresponding mass spectrum. Absorbance was recorded at 254 nm (t_R: 3.57 min). Eluents: solution A (99.9% water, 0.1 % trifluoroacetic acid), solution B (100% acetonitrile). Gradient: 5% to 100% B in 5 min, then up to 10 min at 100% B. Flow rate: 0.5 mL/min.

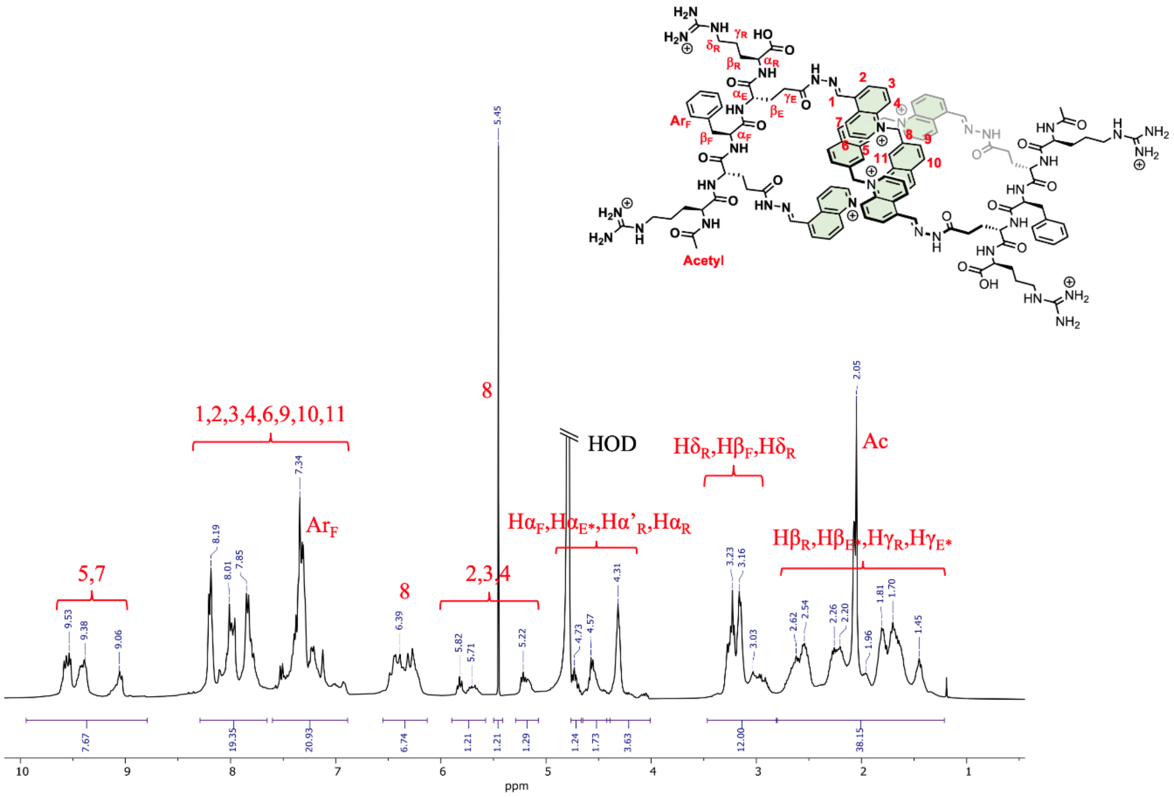

Figure S11: ^{1}H NMR spectrum of **Cat-a** (D_2O, 298 K).

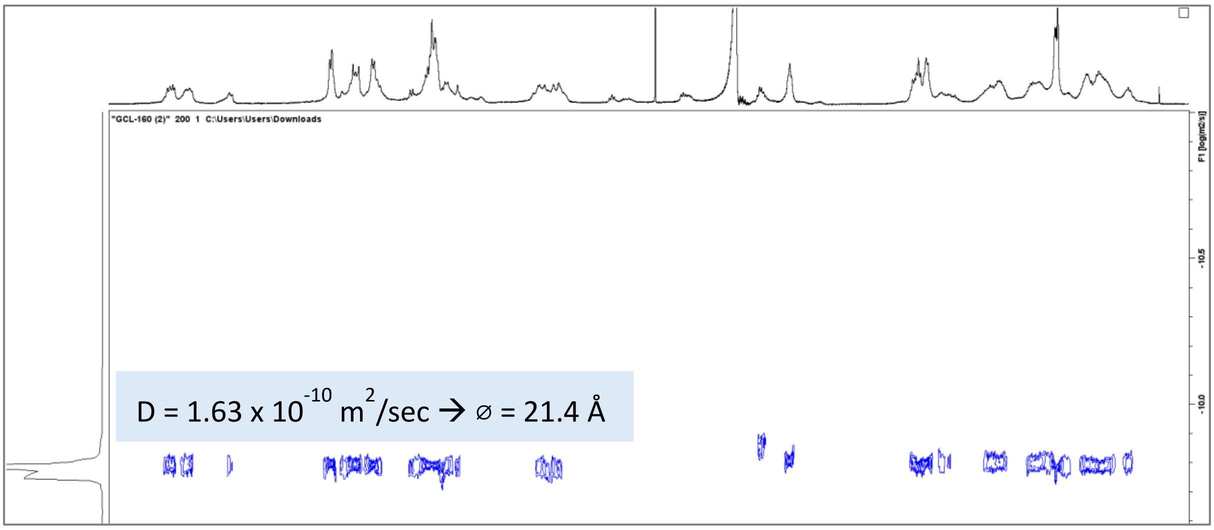

Figure S12: DOSY NMR spectrum of **Cat-a** (D_2O, 298 K).

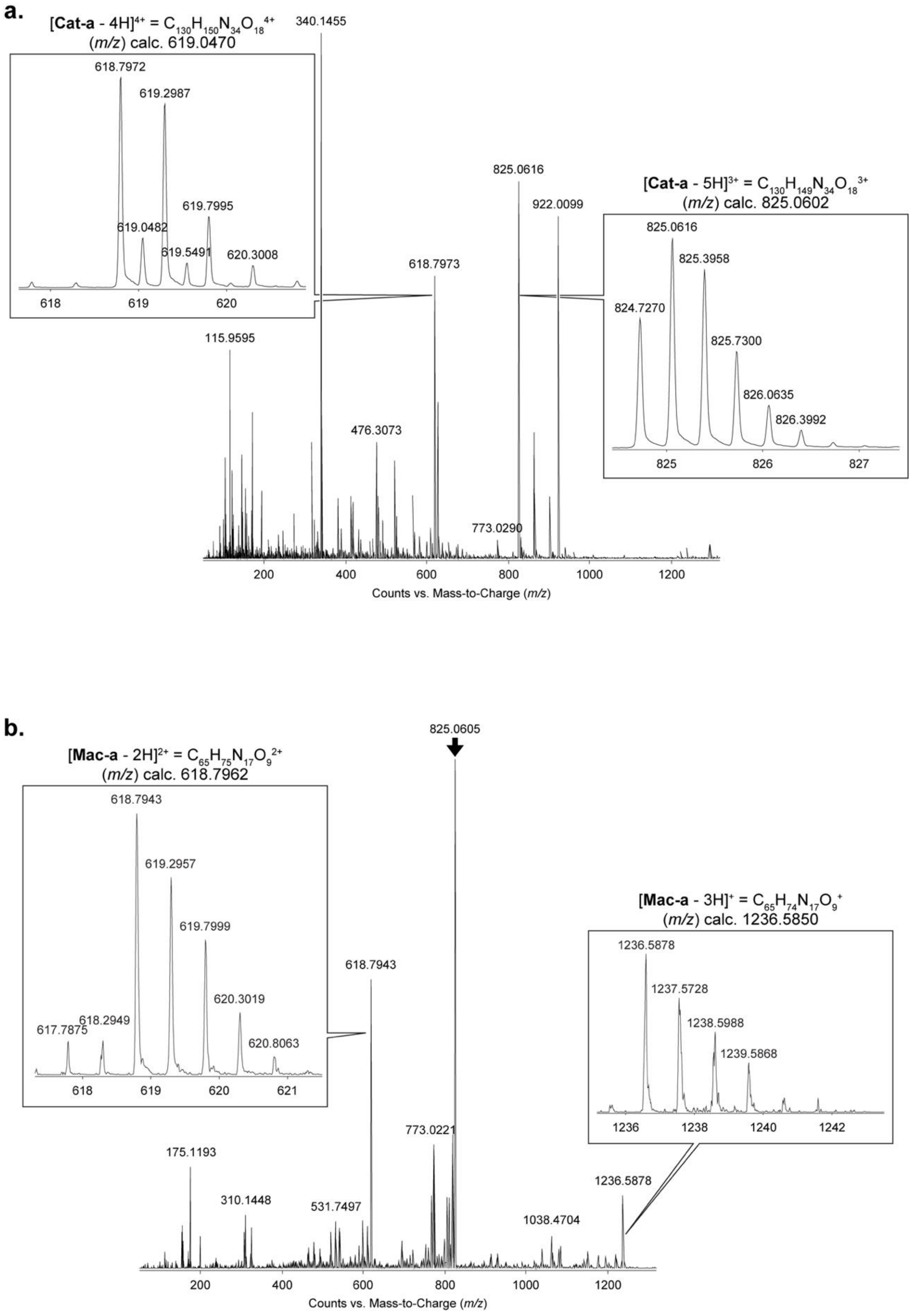

Figure S13. (a) HR-MS and (b) MS/MS fragmentation of **Cat-a** (precursor ion: *m/z* 825, collision energy voltage: 20 V).

Mac-b and Cat-b. Macrocycle **Mac-b** and [2]catenane **Cat-b** were synthesized according to the general procedure and purified by preparative HPLC. Eluents: solution A (99.9% water, 0.1 % trifluoroacetic acid), solution B (99.9% acetonitrile, 0.1 % trifluoroacetic acid). Gradient: 0 min, 5% B; 1 min, 5% B; 15 min, 100% B. Flow rate: 12 mL/min. Yield: 0.76 mg (**Mac-b**) and 1.29 mg (**Cat-b**) (2% each). HR-ESI-MS *m/z* calc. for $[C_{56}H_{68}N_{16}O_8\text{-}2H]^{2+}$ 545.2620, found 545.2598; $[C_{56}H_{68}N_{16}O_8\text{-}3H]^{+}$ 1089.5166, found 1089.5184; $[C_{112}H_{136}N_{32}O_{16}\text{-}5H]^{3+}$ 727.0146 found 727.0154.

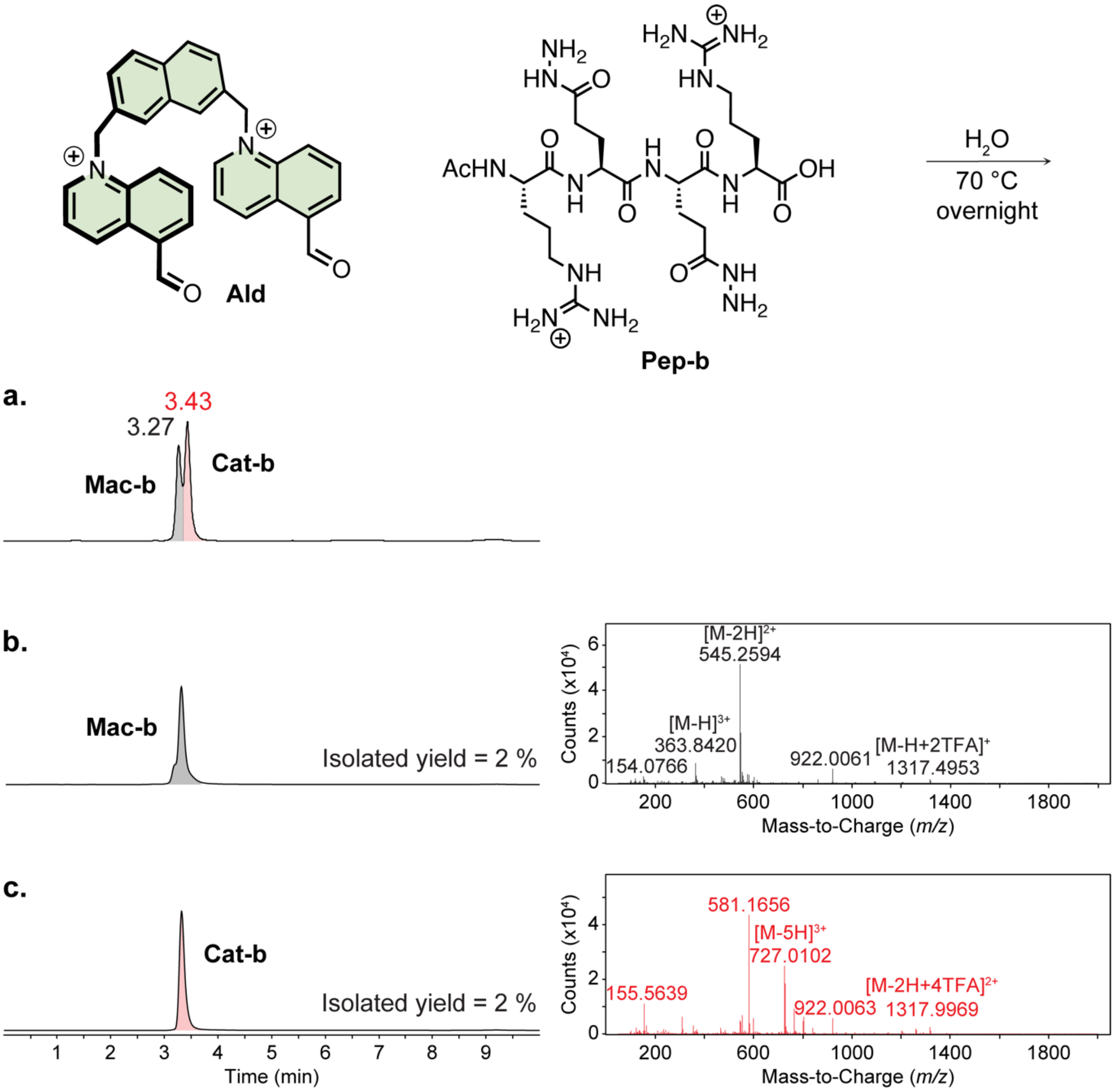

Figure S14. UHPLC-MS analysis of (a) the crude reaction mixture generated from **Ald** and **Pep-b**, (b) isolated **Mac-b** (isolated yield: 2%), and (c) isolated **Cat-b** (isolated yield: 2%). Absorbance was recorded at 254 nm (t_R: 3.27 min for **Mac-b** and 3.43 min for **Cat-b**). Eluents: solution A (99.9% water, 0.1 % trifluoroacetic acid), solution B (100% acetonitrile). Gradient: 5% to 100% B in 5 min, then up to 10 min at 100% B. Flow rate: 0.5 mL/min. The mass spectrum of the isolated compounds are presented next to each chromatogram. The isolated yields are particularly low because it proved difficult to obtain a good separation between **Mac-b** and **Cat-b** during purification.

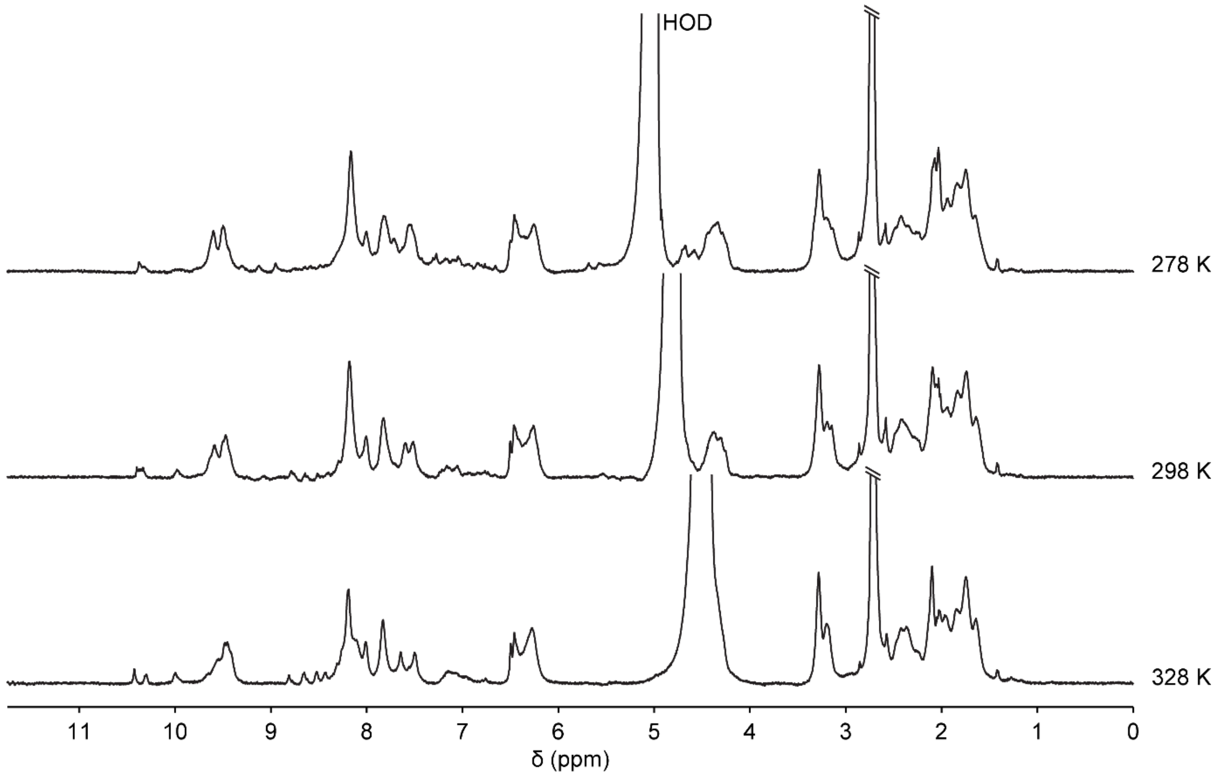

Figure S15. ^{1}H NMR spectrum of **Cat-b** (D_2O, 500 MHz) at variable temperature.

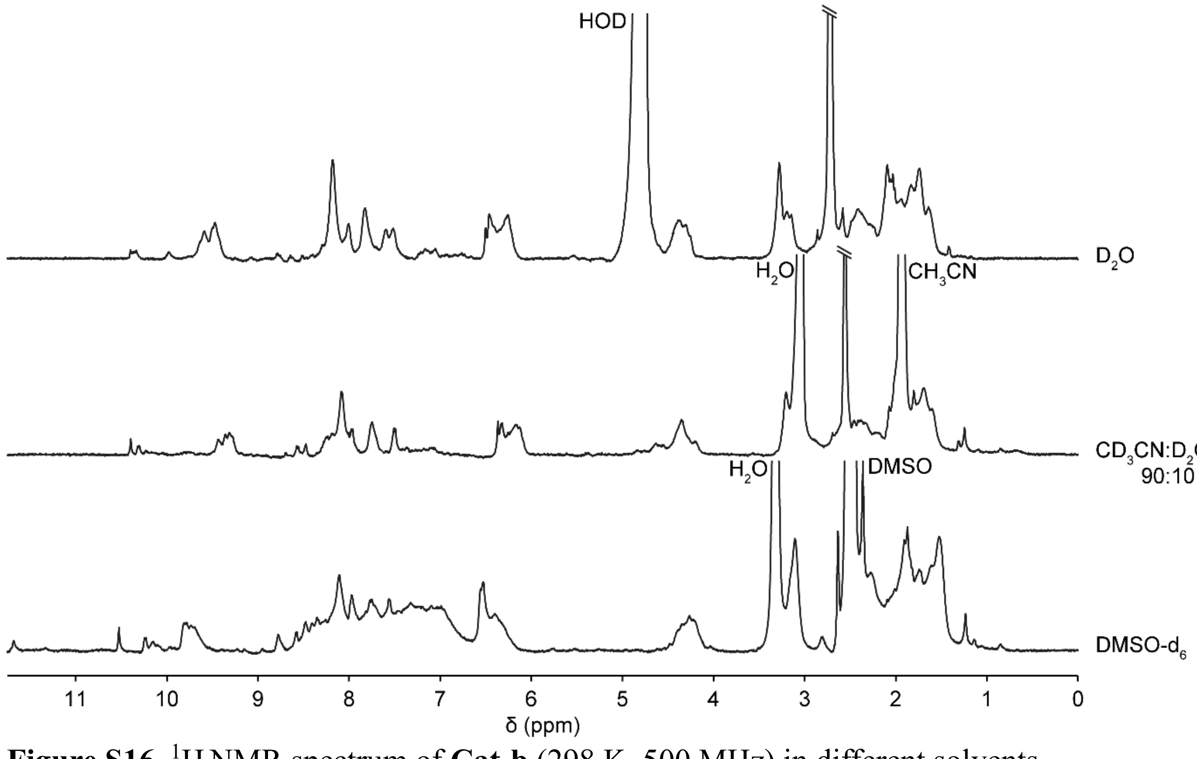

Figure S16. ^{1}H NMR spectrum of **Cat-b** (298 K, 500 MHz) in different solvents.

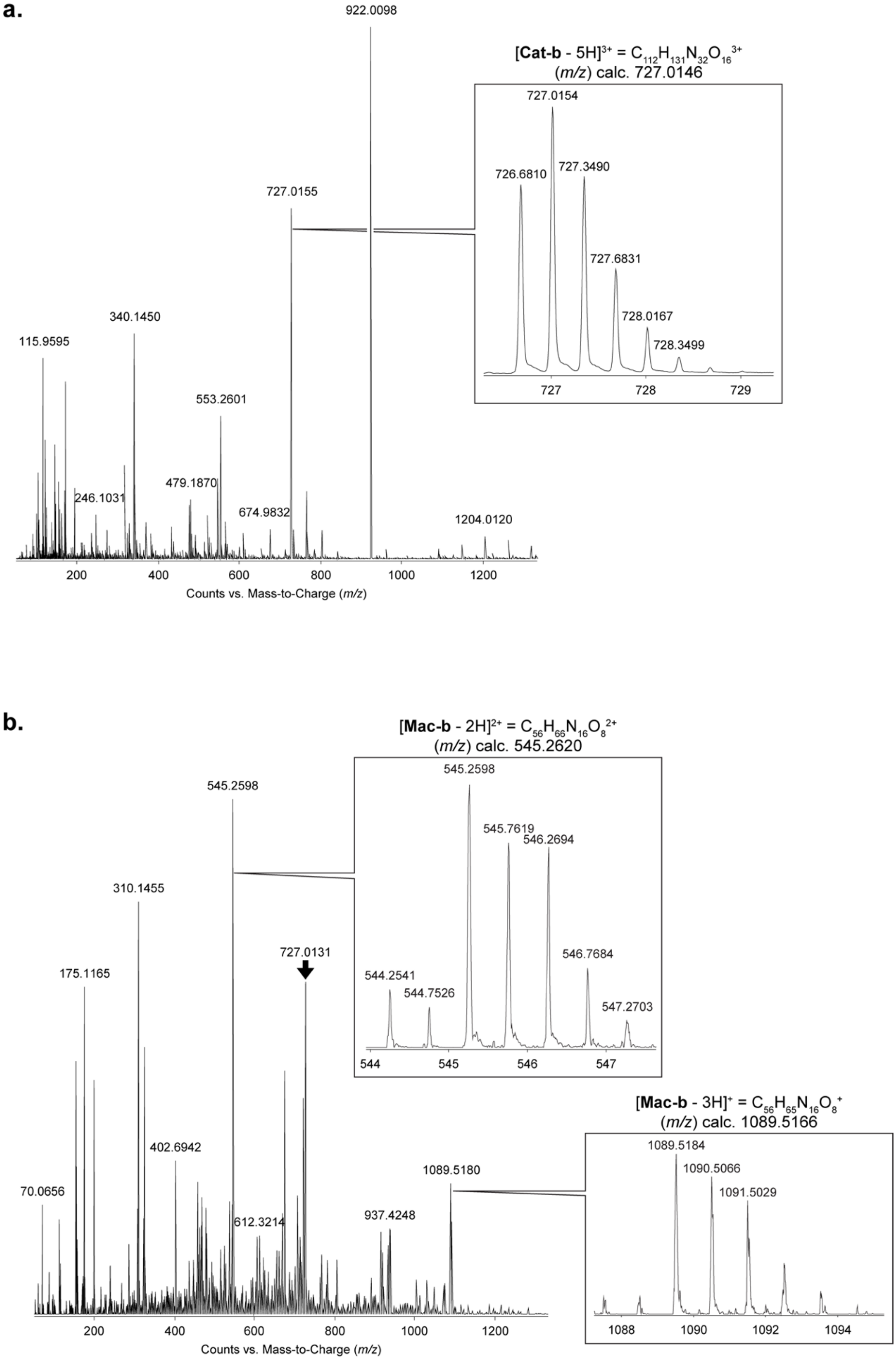

Figure S17. (a) HR-MS and (b) MS/MS fragmentation of **Cat-b** (precursor ion: *m/z* 727, collision energy voltage: 30 V).

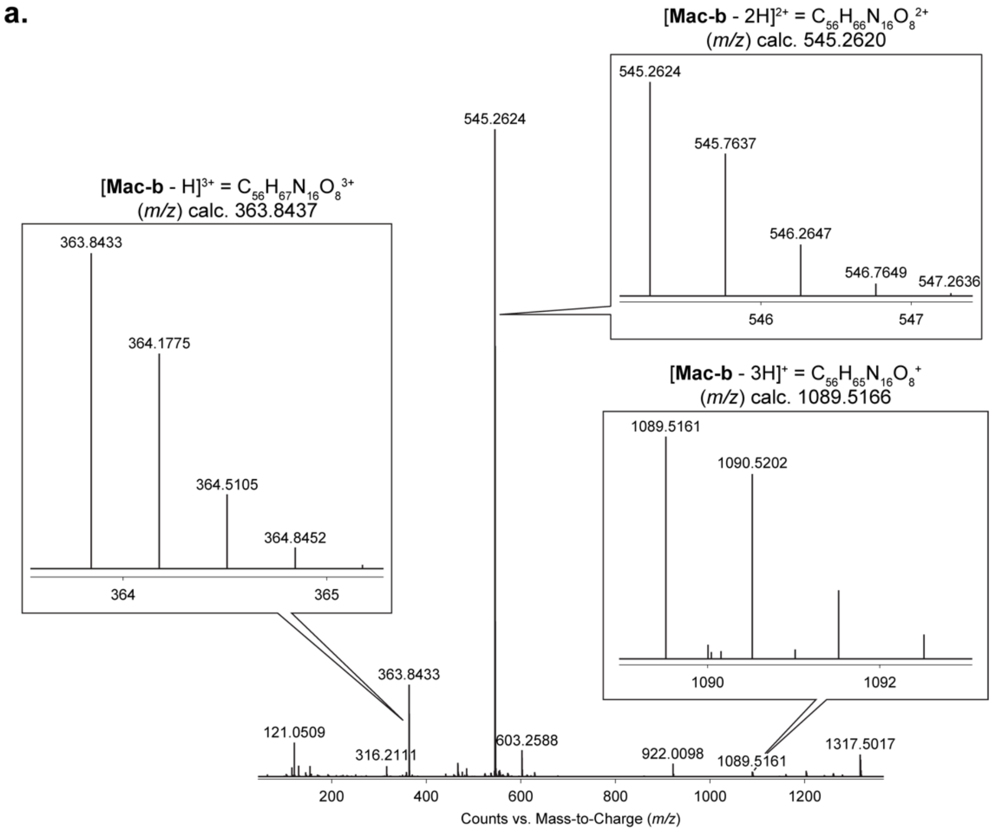

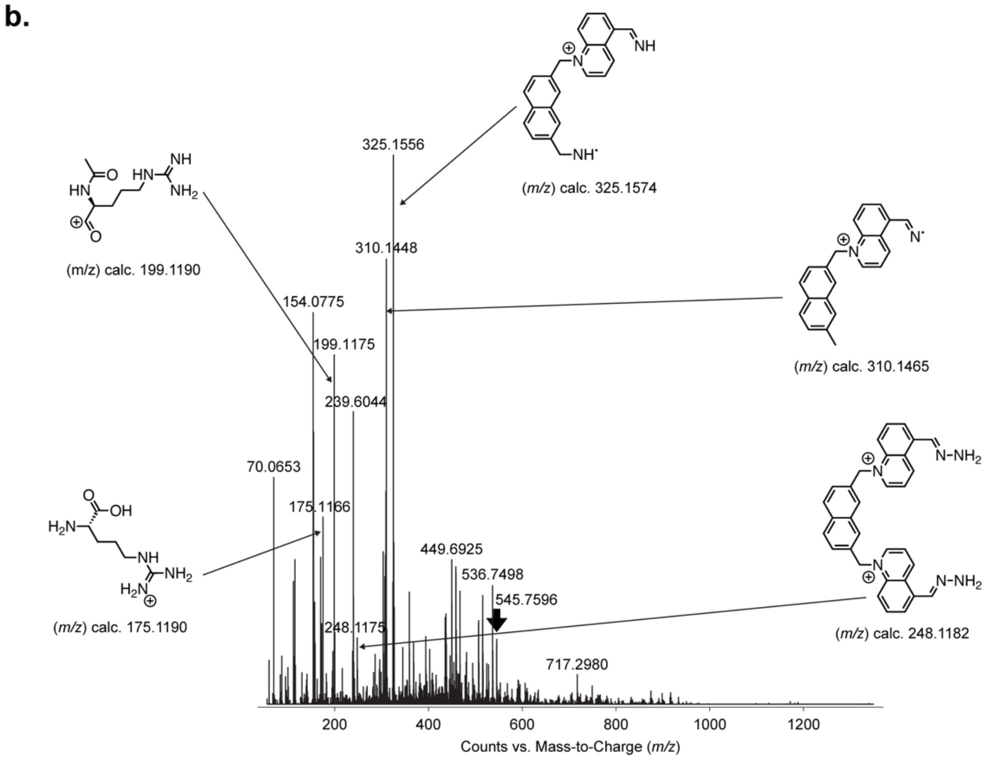

Figure S18. (a) HR-MS and (b) MS/MS fragmentation of **Mac-b** (precursor ion: *m/z* 545, collision energy voltage: 30 V).

4 Protease Assays

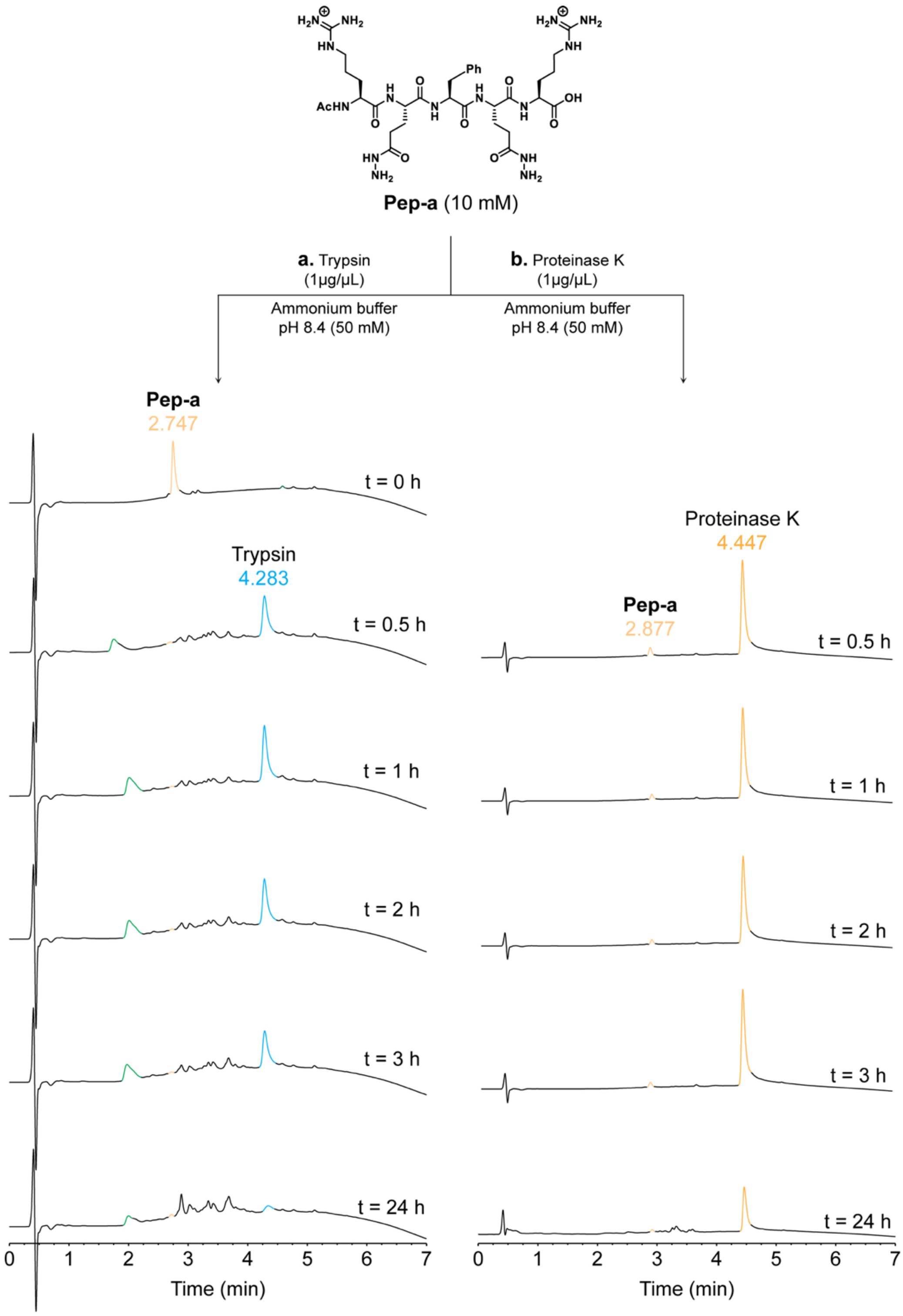

Figure S19. Analytical HPLC traces (214 nm) of **Pep-a** measured at different times after addition of (a) trypsin and (b) proteinase K. Eluents: solution A (99.9% water, 0.1 % trifluoroacetic acid), solution B (99.9% acetonitrile, 0.1 % trifluoroacetic acid). Gradient: 5 to 95 % B in 7 min. Flow rate: 0.5 mL/min.

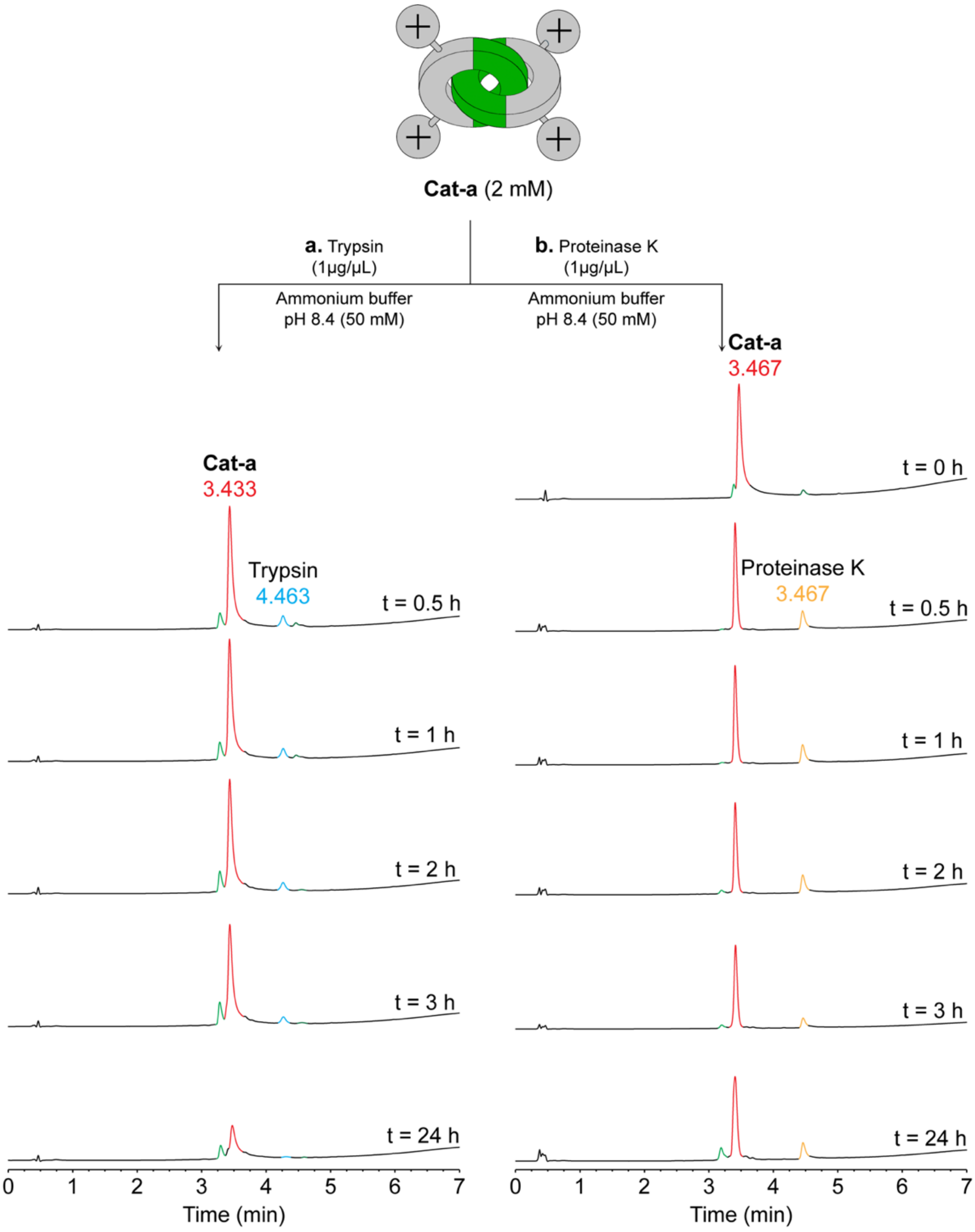

Figure S20. Analytical HPLC traces (254 nm) of **Cat-a** measured at different times after addition of (a) trypsin and (b) proteinase K. Eluents: solution A (99.9% water, 0.1 % trifluoroacetic acid), solution B (99.9% acetonitrile, 0.1 % trifluoroacetic acid). Gradient: 5 to 95 % B in 7 min. Flow rate: 0.5 mL/min.

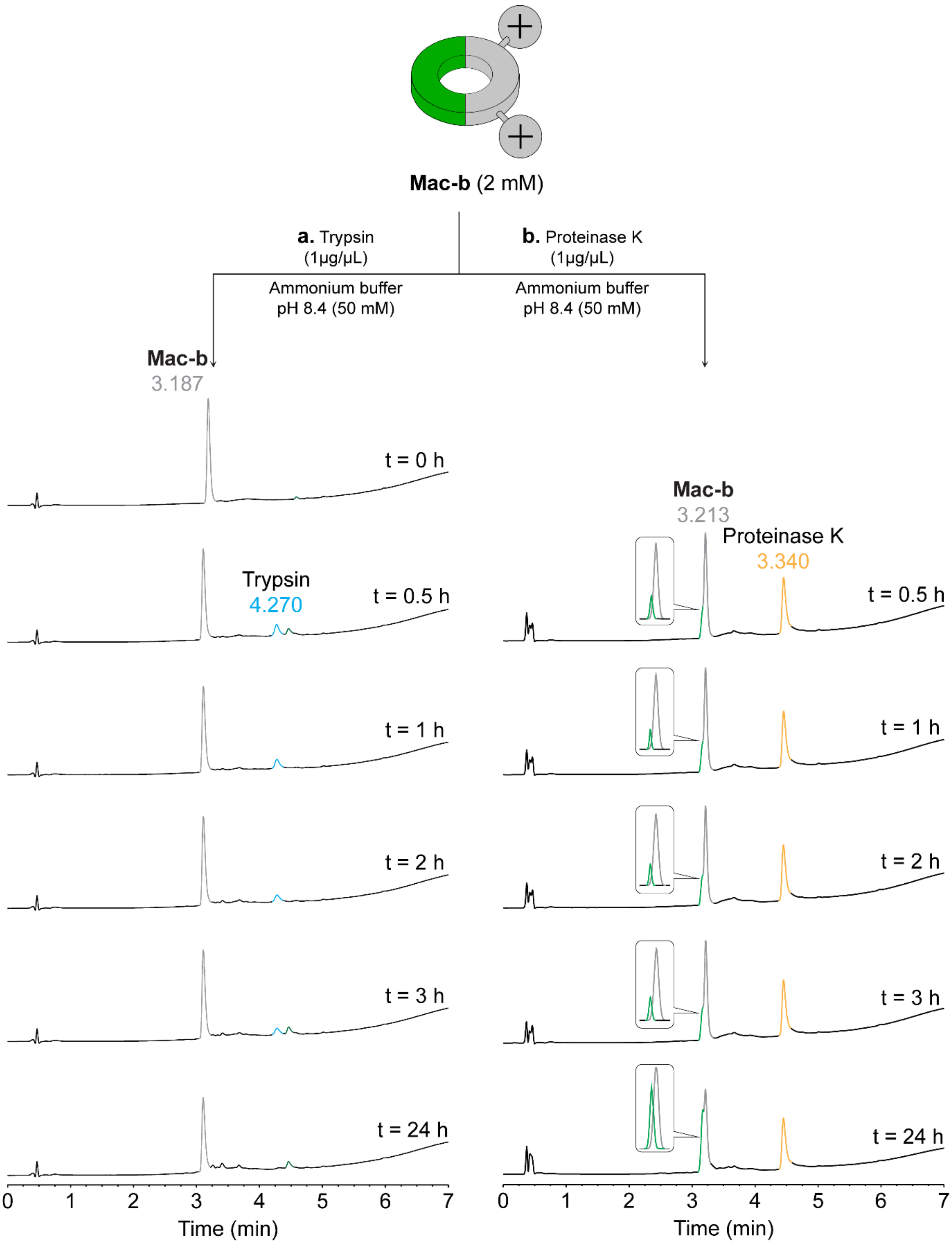

Figure S21. Analytical HPLC traces (254 nm) of **Mac-b** measured at different times after addition of (a) trypsin and (b) proteinase K. Eluents: solution A (99.9% water, 0.1 % trifluoroacetic acid), solution B (99.9% acetonitrile, 0.1 % trifluoroacetic acid). Gradient: 5 to 95 % B in 7 min. Flow rate: 0.5 mL/min.

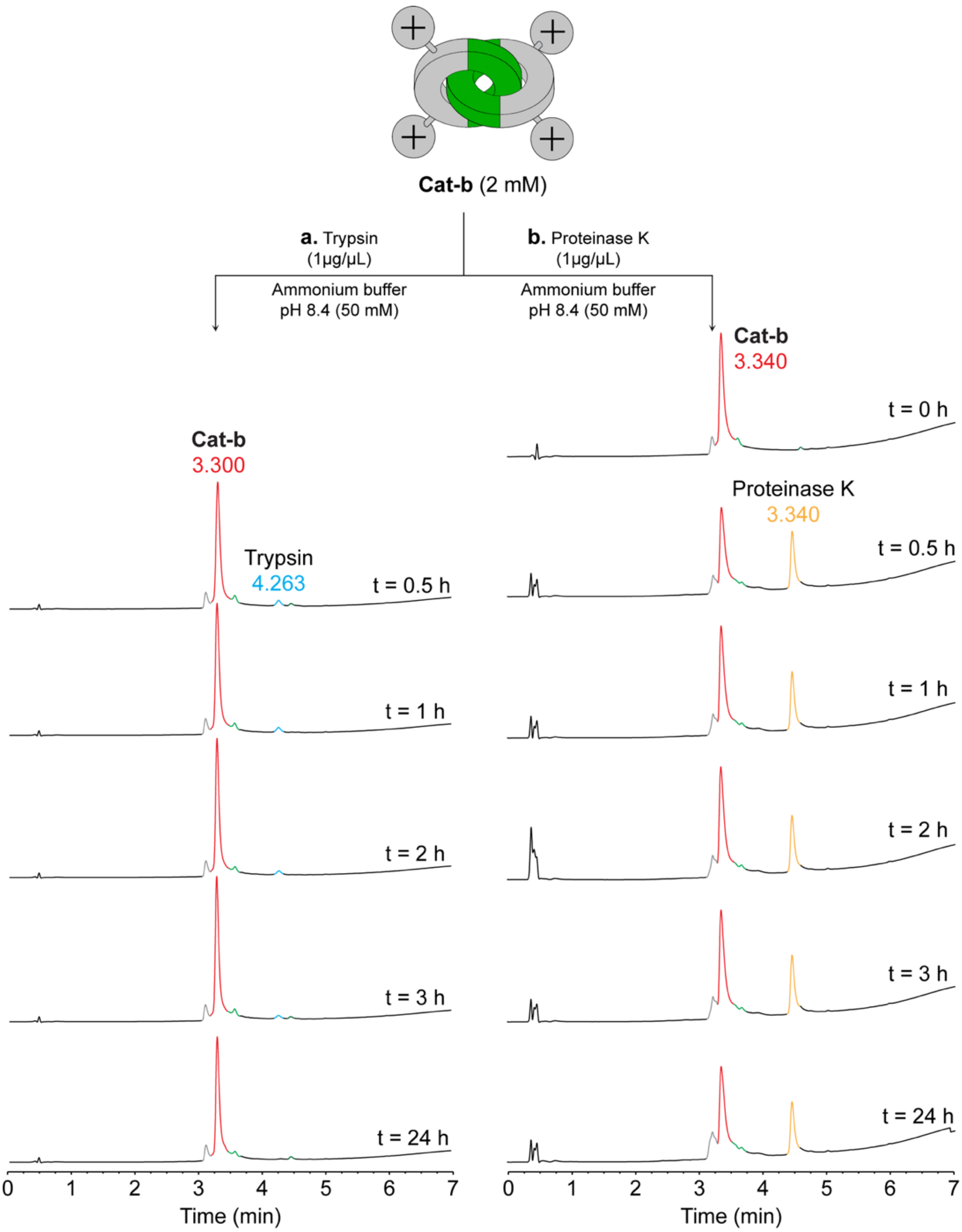

Figure S22. Analytical HPLC traces (254 nm) of **Cat-b** measured at different times after addition of (a) trypsin and (b) proteinase K. Eluents: solution A (99.9% water, 0.1 % trifluoroacetic acid), solution B (99.9% acetonitrile, 0.1 % trifluoroacetic acid). Gradient: 5 to 95 % B in 7 min. Flow rate: 0.5 mL/min.

5 Gel electrophoresis assays

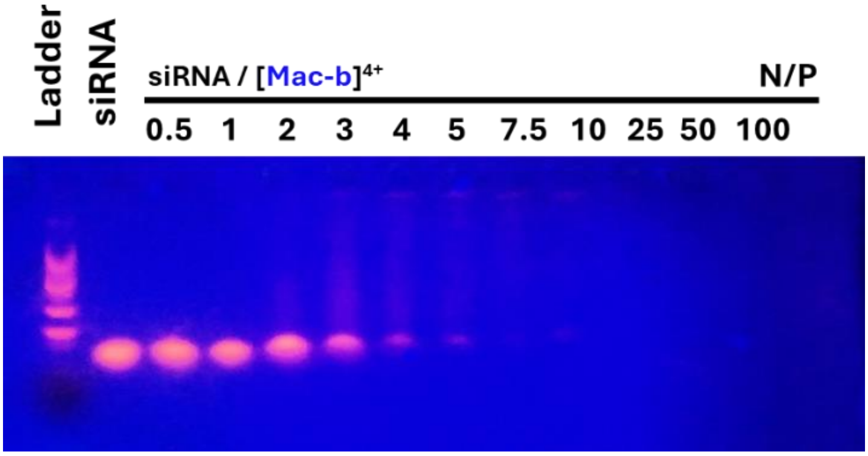

Figure S23. Gel electrophoresis siRNA complexation assay showing complexation capability of **Mac-b**. Assay was performed by incubation of the different compounds with siRNA in PBS buffer (25 mM, 150 mM NaCl, pH 7.2) for 30 min at room temperature.

6 Dynamic light scattering

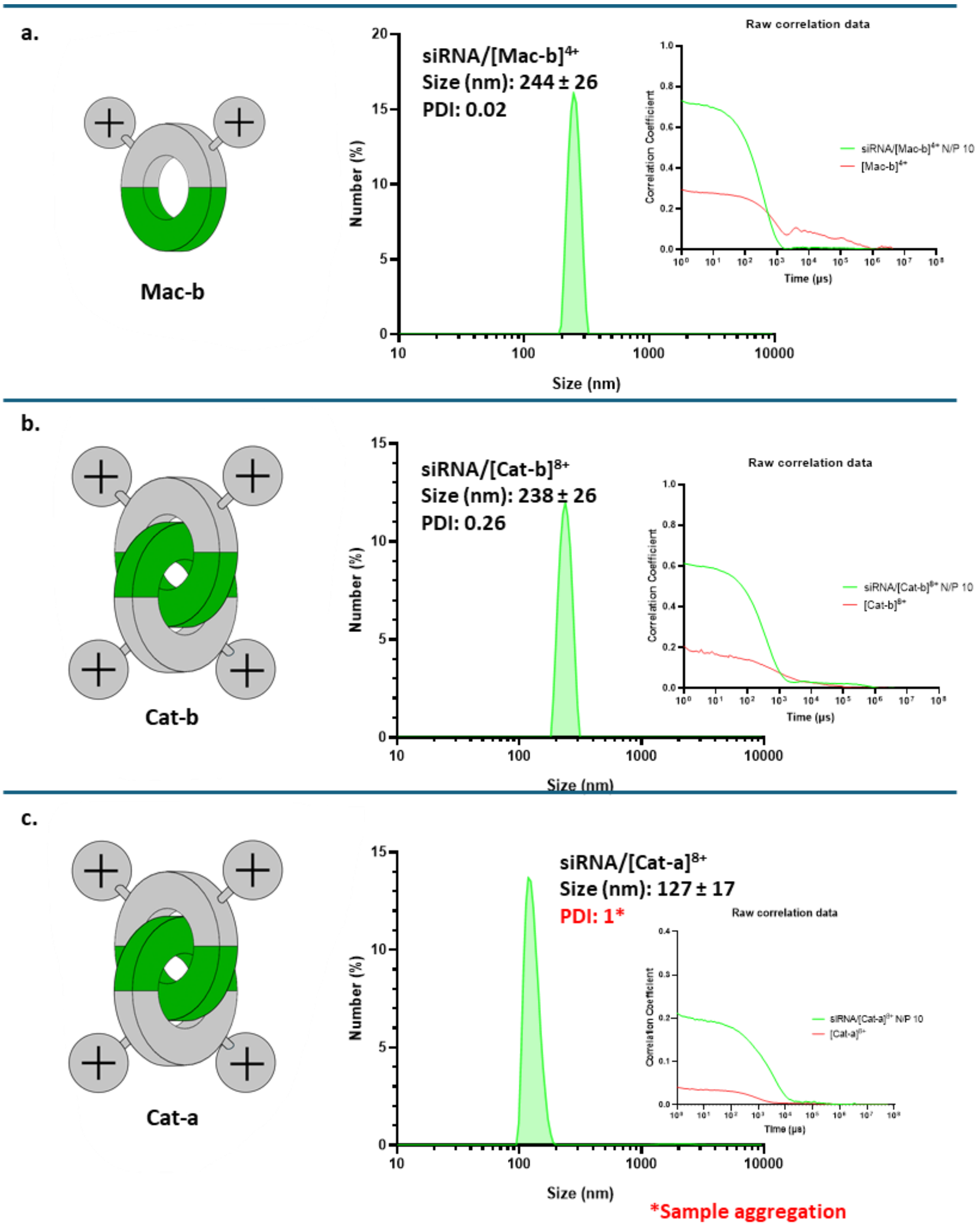

Figure S24. Dynamic light scattering analysis of the siRNA complexes formed with (a) **Mac-b**, (b) **Cat-b** and (c) **Cat-a**. All samples were preapred at N/P 10 (green raw correlation data line). Non-containing siRNA counterparts were also analysed at the same concentration (red raw correlation data line). Size distribution was then observed by number. Analysis (c) showed sample aggregation which translated in a high PDI value.

7 References

[1] K. Caprice, D. Pál, C. Besnard, B. Galmés, A. Frontera, F. B. L. Cougnon, "Diastereoselective Amplification of a Mechanically Chiral [2]Catenane" *J. Am. Chem. Soc.* **2021**, *143*, 11957-11962.

[2] J. García-Coll, P. Trousselier, S. D. Pawar, Y. Bessin, L. Lichon, J. L. Chain, E. Sachon, N. Bettache, S. Ulrich, "Amphiphilic Dynamic Covalent Polymer Vectors of Sirna" *Chem. Sci.* **2025**, *16*, 2413-2419.

[3] E. Bartolami, Y. Bessin, V. Gervais, P. Dumy, S. Ulrich, "Dynamic Expression of DNA Complexation with Self-Assembled Biomolecular Clusters" *Angew. Chem. Int. Ed.* **2015**, *54*, 10183-10187.

[4] J. Garcia-Coll, L. M. A. Ali, J. Montenegro, N. Bettache, S. Ulrich, "Mrna Delivery with Templated Dynamic Covalent Polymers" *Chem. Commun.* **2025**, *61*, 4050-4053.

[5] B. D. Gonzalez, R. Lopez-Blanco, S. Parcero-Bouzas, N. Barreiro-Piñeiro, L. Garcia-Abuin, E. Fernandez-Megia, "Dynamic Covalent Boronate Chemistry Accelerates the Screening of Polymeric Gene Delivery Vectors Via Complexation of Nucleic Acids" *J. Am. Chem. Soc.* **2024**, *146*, 17211-17219.

[6] E. R. Gillies, F. Deiss, C. Staedel, J. M. Schmitter, I. Huc, "Development and Biological Assessment of Fully Water-Soluble Helical Aromatic Amide Foldamers" *Angew. Chem. Int. Ed.* **2007**, *46*, 4081-4084.
